# Protocol for Cas9 nickase-mediated induction and two-dimensional gel detection of i-loops at human telomeric and alpha satellite repeats

**DOI:** 10.1016/j.xpro.2026.104888

**Published:** 2026-10-06

**Authors:** Elia Zanella, Flavia Bruno, Ylli Doksani

**Affiliations:** 1 IFOM ETS – The AIRC Institute of Molecular Oncology, Via Adamello 16, 20139 Milan, Italy

**Keywords:** Cell Biology, Cancer, Molecular Biology, CRISPR

## Abstract

Single-stranded DNA breaks (SSBs) at repetitive elements promote internal loops (i-loops), precursors of extrachromosomal circular DNA (eccDNA). Here, we present a protocol for inducing targeted breaks at human telomeric or alpha-satellite repeats using doxycycline-inducible Cas9 nickase variants and repeat-specific sgRNAs. We describe steps for verifying strand-specific nicking by alkaline-gel Southern blotting and detecting i-loop accumulation by two-dimensional agarose gel electrophoresis (2D gel). In this protocol, DNase I treatment of isolated nuclei is employed as a Cas9-independent control.

For complete details on the use and execution of this protocol, please refer to Zanella et al.^1^

## Highlights

• Steps for generating doxycycline-inducible Cas9 nickase human cell lines
• Instructions for doxycycline-induced nicking at telomeric and alpha-satellite repeats
• Steps for verifying strand-specific nicks by alkaline gel electrophoresis
• Detection of i-loops by two-dimensional gel electrophoresis and Southern blotting

## Abstract (editor highlights)

Publisher’s note: Undertaking any experimental protocol requires adherence to local institutional guidelines for laboratory safety and ethics.

## Graphical abstract

**Figure:**
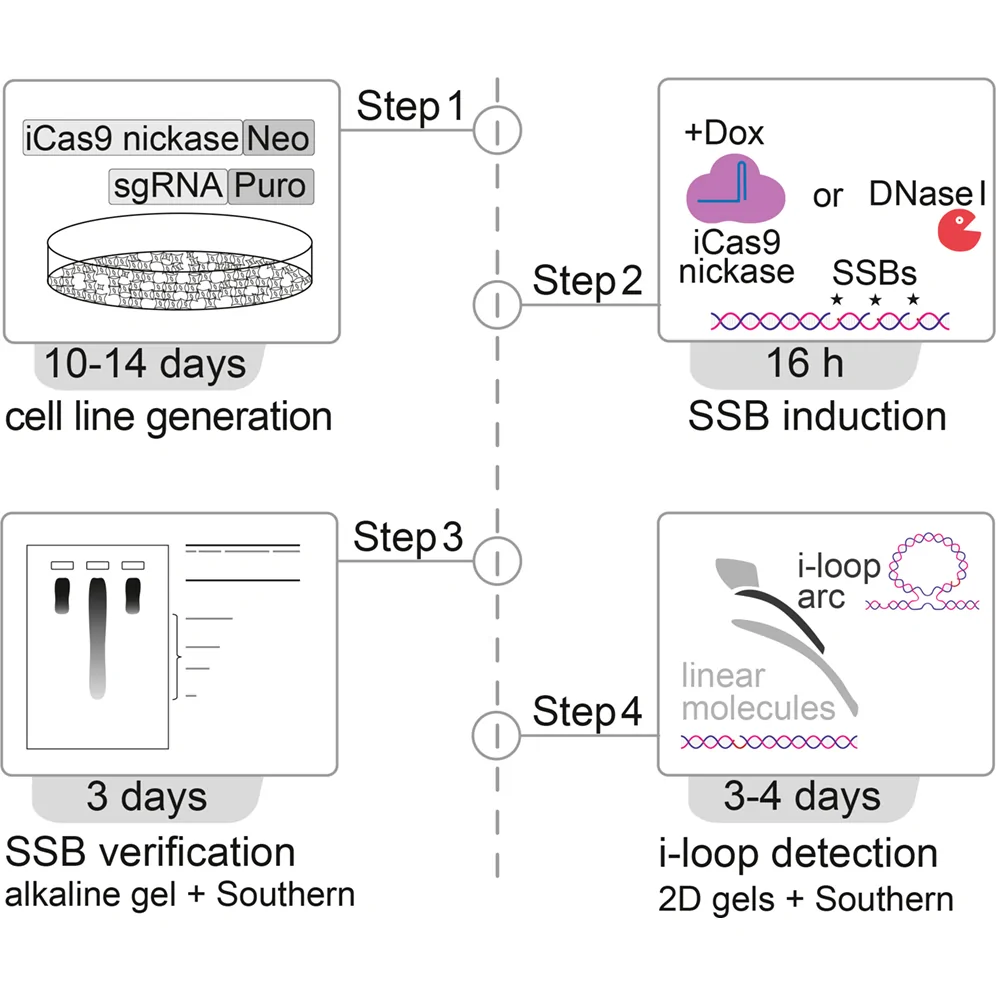

## Before you begin

The protocol below describes the specific steps used with HT1080 human fibrosarcoma cells. However, the doxycycline-inducible Cas9 nickase system and the downstream 2D-gel analysis can be adapted to other human cell lines that support stable lentiviral integration, antibiotic selection and Tet-On-regulated transgene expression. Antibiotic selection concentrations, doxycycline induction conditions and basal Cas9 nickase leakiness should be empirically optimized for each cell line before proceeding to 2D-gel analysis. The procedure described in “DNase I treatment of isolated nuclei for nick induction (Cas9-independent control method)”, steps 16–25 provides a Cas9-independent control for nick-induced i-loop formation and can be applied to isolated nuclei from adherent or suspension cell lines.

### Innovation

This protocol describes a two-component lentiviral approach that separates doxycycline-inducible Cas9 nickase expression from constitutive sgRNA delivery. Unlike chemical nick-inducing agents, this system allows targeted induction of SSBs at user-defined repetitive sequences. In the applications described here, it enables comparison of i-loop accumulation between the targeted repeat locus and a non-targeted repeat in the same cell population and at the same time point, providing an internal control for locus-specific i-loop induction. At certain tandem repeats, such as telomeres, the orientation of the sgRNA relative to the repeat sequence can be exploited to achieve strand-specific nicking.^2^^,^^3^^,^^4^ The telomeric sgRNA pairs with the C-rich strand, allowing the two Cas9 nickase variants, Cas9^D10A^ and Cas9^H840A^, to introduce nicks on opposite DNA strands.^5^^,^^6^^,^^7^ As a Cas9-independent control, the DNase I treatment described in steps 16–25 provides an alternative way to induce nicking and i-loop formation, although it does not provide locus or strand specificity.

### Institutional permissions

Any experiments involving genetically modified human cell lines and lentiviral vectors must be performed in accordance with relevant institutional and national biosafety guidelines. Before beginning this protocol, obtain approval from the appropriate institutional biosafety committee or equivalent regulatory body, for the generation and use of replication-incompetent lentiviral vectors, under the required biosafety containment conditions. All work with radioactive materials, including γ-^32^P-ATP and α-^32^P-dCTP must be conducted in a licensed radiation work area using appropriate beta shielding and personal protective equipment, as required by local radiation safety regulations.

### Mutagenic PCR and plasmid preparation

**Timing: 1–2 weeks**

This section describes the generation of Cas9^D10A^ and Cas9^H840A^ nickase starting from the parental doxycycline-inducible Cas9 expression vector and the cloning of repeat-targeting sgRNAs into the lentiGuide-Puro backbone. When targeted to telomeres with a G-rich sgRNA, the two Cas9 nickase variants cleave opposite DNA strands: Cas9^D10A^ nicks the target strand (i.e., the telomeric C-strand, which pairs with the sgRNA) while Cas9^H840A^ nicks the non-target strand (i.e., the telomeric G-strand). The expected products of this section are four sequence-verified, endotoxin-free maxiprep plasmids: lenti-iCas9^D10A^-Neo, lenti-iCas9^H840A^-Neo, Teloguide and Alphaguide, which are the inputs for lentivirus production. Together with the two transductions that follow, this work constitutes Step 1 of the graphical abstract.

1. Obtain the parental Lenti-iCas9-Neo plasmid (Addgene #85400) encoding doxycycline-inducible, FLAG-tagged wild-type Cas9. Also obtain the lentiGuide-Puro plasmid (Addgene #52963) used here as the sgRNA expression backbone.^5^
2. Introduce the D10A mutation into the iCas9 backbone by two parallel single-primer extension reactions (a SPRINP-type site-directed mutagenesis strategy)^6^ to generate the Cas9^D10A^ nickase variant.
  ***Note:*** Unlike conventional two-primer PCR, each reaction contains a single mutagenic primer and therefore produces linear, single-stranded amplification products rather than exponentially amplified double-stranded product. The two reactions are pooled and denatured/reannealed in step 2c so that complementary mutant strands from the two reactions hybridize to form the nicked, mutation-bearing circular plasmid that is recovered after DpnI digestion. This design avoids the primer-dimer and template-concatemer artifacts associated with running both mutagenic primers in a single reaction on a large (∼14 kb) lentiviral plasmid.
  a. Prepare two single-primer extension reactions, each 50 μL, containing 500 ng plasmid template, Q5 polymerase buffer 1×, 0.02 U/μL of Q5 polymerase, 200 μM each dNTP and 0.5 μM of one mutagenic primer. For reaction 1, use Cas9^D10A^ mutagenic FW; for reaction 2, use Cas9^D10A^ mutagenic RV. Do not combine the two primers in a single tube.
  b. Set up the thermocycler as follows: 1 hold at 98°C for 30 s; 30 cycles of 98°C for 10 s, 64°C for 20 s, 72°C for 12 min (45 sec/kb); 1 hold at 72°C for 2 min.
  c. After cycling, pool the two reactions into one tube. Heat to 98°C for 5 min, then cool slowly to 20–25°C (approximately 0.1°C/s, or by switching the block off and leaving the tube in place for 45–60 min) to allow annealing of complementary mutant strands.
  d. Add 30 U of DpnI to the pooled reaction. Incubate at 37°C 12–16 h to digest the methylated parental plasmid template.
  e. Transform 5 μL of the DpnI-digested product into *E. coli*, Stbl3 competent cells.
    i. Plate on LB-agar containing 100 μg/mL ampicillin.
    ii. Incubate at 37°C for 12–16 h.
    iii. Pick at least 12 colonies and confirm the D10A mutation by Sanger sequencing.
3. Introduce the H840A mutation into the iCas9 backbone by the same single-primer extension strategy to generate the Cas9^H840A^ nickase variant.
  a. Repeat Step 2 using the Cas9^H840A^ mutagenic FW and Cas9^H840A^ mutagenic RV to generate the H840A variant. Use *E. coli* Stbl3 competent cells for transformation of lentiviral plasmids.
4. Clone the repeat-targeting sgRNAs into the BsmBI-digested lentiGuide-Puro transfer plasmid to generate Teloguide and Alphaguide plasmids, which harbor the telomere-specific and alpha-satellite-specific sgRNAs, respectively.^1^^,^^7^^,^^8^
  a. Phosphorylate and anneal oligonucleotides: For each target, combine the forward and reverse oligonucleotides with T4 polynucleotide kinase (PNK) in T4 ligation buffer 1×.
    **CRITICAL:** Use T4 ligation buffer instead of T4 PNK buffer, as the ligation buffer provides the ATP required for the phosphorylation reaction.
  b. Incubate the mixture in a thermocycler using the following parameters to simultaneously phosphorylate and anneal the oligonucleotides:
     37°C for 30 min.
     95°C for 5 min.
     Ramp down to 25°C at a rate of 5°C/min.
  c. Dilute the annealed oligonucleotide duplexes 1:200 in sterile water.
  d. Ligate into the backbone.
    i. Mix 50 ng of BsmBI-digested lentiGuide-Puro plasmid with 1 μL of the diluted oligo duplex in 2× Quick Ligase Buffer with Quick Ligase (NEB).
    ii. Incubate the reaction at 20–25°C for 10 min.
    iii. *Set up* a parallel negative-control ligation using water instead of the oligo duplex.
  e. Transform the ligation products into recombination-deficient E. coli competent cells (such as Stbl3) to prevent unwanted recombination of the viral long-terminal repeats. Sequence-verify the U6-sgRNA insertion site in all selected constructs before proceeding.
    ***Note:*** The negative-control ligation should yield at least tenfold fewer colonies than the duplex-containing ligation. A high background indicates incomplete BsmBI digestion or incomplete removal of the filler fragment from the backbone.
5. Scale up all four sequence-verified constructs, lenti-iCas9^D10A^-Neo, lenti-iCas9^H840A^-Neo, Teloguide and Alphaguide by endotoxin-free maxiprep. Elute in 10 mM Tris-HCl pH 8.0. Aim to obtain: ≥500 μg per plasmid at ≥1 μg/μL.

***Note:*** We use the NucleoBond Xtra Maxi kit (Macherey-Nagel) following the manufacturer’s protocol. Any endotoxin-free, anion-exchange or silica-based maxiprep system that yields supercoiled plasmid at ≥1 μg/μL is an acceptable substitute; endotoxin removal is important because residual endotoxin reduces HEK293-T transfection efficiency and therefore viral titer.]

**Pause point:** Maxiprep plasmids can be stored at −20°C for 1–2 years. Thaw on ice and mix by gentle inversion before use. Do not vortex lentiviral plasmids.

### Lentivirus production in HEK293-T cells

**Timing: 3–5 days**

Here we describe production of VSV-G-pseudotyped, third-generation lentiviral particles by transient polyethylenimine (PEI)-mediated transfection of HEK293-T cells. PEI condenses plasmid DNA into positively charged polyplexes that are taken up by endocytosis; the transfer plasmid is co-delivered with three packaging plasmids that supply Gag-Pol (pMDLg/pRRE), Rev (pRSV-Rev) and the VSV-G envelope (pMD2.G). The output of this section is filtered, unconcentrated viral supernatant used directly for the two sequential transductions (Step 1 of the graphical abstract). Lentivirus production is not the focus of this protocol; readers requiring a fully detailed description of vector production, concentration and titration should refer to Kutner et al. (2009).^9^

6. One day before transfection, seed HEK293-T cells at 8 × 10^6^ cells per 15-cm dish in 20 mL DMEM supplemented with 10% Tet-free FBS and 2 mM L-glutamine, without antibiotics. Cells should reach 70%–80% confluency on the day of transfection.

**CRITICAL:** Use Tet-free (tetracycline-free) FBS for all HEK293-T and HT1080 cultures throughout this protocol. Conventional FBS may contain trace tetracyclines that can activate the Tet-On Cas9 system and produce leaky nickase expression, which may confound i-loop detection in uninduced controls.

7. Prepare the PEI-DNA complex per 15-cm dish.
  a. In a sterile 15-mL tube, dilute 30 μg total plasmid DNA, consisting of 15 μg lentiviral transfer plasmid, 4.2 μg pMD2.G, 7.5 μg pMDLg/pRRE and 3.75 μg pRSV-Rev, in 1.5 mL serum-free DMEM and mix by vortexing.
  b. In a second tube, dilute 150 μg PEI Max 40000 in 1.5 mL serum-free DMEM. The PEI:DNA mass ratio is 5:1. Mix by vortexing.
  c. Add the PEI dilution to the DNA dilution, obtaining 3 mL final volume. Mix by gentle vortexing for 5 s.
  d. Incubate for 10 min at 20–25°C to allow polyplex formation. Use within 20 min as polyplexes aggregate and lose activity on longer standing.

***Note:*** PEI is our preferred transfection reagent because of its cost and reproducibility, but it is not essential for lentivirus production. Calcium phosphate co-precipitation or a commercial lipid-based reagent (for example Lipofectamine 3000) can be substituted without changing any downstream steps, provided the resulting supernatant gives comparable transduction efficiency in HT1080 cells.

8. Add the PEI-DNA complex dropwise to the HEK293-T dish, distributing it evenly across the surface, and rock the dish gently.
9. Replace the medium 8–12 h post-transfection to reduce PEI cytotoxicity.
  a. Aspirate gently the transfection medium.
  b. Add 20 mL of fresh DMEM supplemented with 10% Tet-free FBS and 2 mM L-glutamine, without antibiotics.

**CRITICAL:** The 20 mL volume added here defines the volume and therefore the effective titer of the viral supernatant used in all subsequent transductions. If a different volume is used, the transduction volumes in steps 2 and 7 of “generation of doxycycline-inducible Cas9 nickase cell lines” must be adjusted proportionally, or the supernatant must be re-titrated.

10. Collect the viral supernatant 36 h post-transfection. The expected recovery is 18–19 mL per 15-cm dish.
  a. Clarify the supernatant by centrifugation at 500 × g for 5 min at 4°C to remove detached producer cells.
  b. Filter immediately through a 0.45-μm low-protein-binding syringe filter. Do not use 0.22-μm filters, which retain a substantial fraction of viral particles.
  c. Use the fresh supernatant directly to infect target cells, or dispense into single-use aliquots and store at −80°C. We store 15-mL aliquots for 15-cm-dish transductions and 5-mL aliquots for 10-cm-dish transductions. Expect a 2- to 4-fold loss of functional titer after one freeze-thaw cycle; do not refreeze.
  d. Add polybrene to 8 μg/mL final concentration, right before use.

***Note:*** A second supernatant collection at 60 h post-transfection can be pooled with the 36-h harvest to increase total titer, but the 36-h supernatant alone typically yields sufficient transduction efficiency for HT1080 cells in our hands.

***Note:*** Estimate the functional titer of a new viral batch, by transducing with a serial dilution of supernatant (for example 1:1, 1:5, 1:25 and 1:125 in complete medium) in a 6-well plate and counting the fraction of cells surviving antibiotic selection. Knowing the titer allows the supernatant to be diluted to a defined multiplicity of infection and removes the need to repeat full-scale killing controls at every subsequent selection (see step 8 of “generation of doxycycline-inducible Cas9 nickase cell lines”).

### Cell culture setup

**Timing: 3–5 days**

This section defines the culture conditions and the quality-control checks that apply to the recipient cell line throughout the protocol. The output is a mycoplasma-free, authenticated HT1080 culture at the correct density for the first lentiviral transduction.

11. Culture HT1080 cells in DMEM supplemented with 10% Tet-free FBS and 2 mM L-glutamine at 37°C in a humidified incubator with 5% CO_2_. Omit penicillin/streptomycin during transduction to avoid reducing transduction efficiency.

***Note:*** These culture conditions are maintained throughout the experiment unless stated otherwise.

12. Verify that all cell lines are mycoplasma-free using the MycoAlert Detection Kit and PCR-based testing upon every thaw and at regular intervals, at least quarterly. Authenticate HT1080 cells by STR profiling, for example using GenePrint 10, before starting experiments.
13. Seed HT1080 cells 24 h before lentiviral transduction to reach 40%–50% confluency on the day of infection. Cells must not exceed 70% confluency at the time of transduction.

## Key resources table

**Table:** 

REAGENT or RESOURCE	SOURCE	IDENTIFIER
**Antibodies**		

Anti-FLAG (mouse monoclonal, clone M2); used at 1:1000	Millipore	Cat# F1804; RRID: AB_262044
Anti-GAPDH (mouse monoclonal), used at 1:5000	Sigma-Aldrich	Cat# G8795; RRID: AB_1078991
Anti-mouse IgG, HRP-linked (horse polyclonal); used at 1:5000	Cell Signaling Technology	Cat# 7076; RRID: AB_330924
Anti-Tubulin (mouse monoclonal); used at 1:5000	Thermofisher	Cat# A11126; RRID: AB_221538

**Bacterial and virus strains**		

E. coli One Shot TOP10 chemically competent cells	Invitrogen	Cat# C4040-03
E. coli One Shot Stbl3 chemically competent cells	Invitrogen	Cat# C737303
Lentiviral particles: Lenti-iCas9-neo (parent backbone)	Addgene	Cat# 85400; RRID: Addgene_85400
Lentiviral particles: LentiGuide-Puro (sgRNA backbone)	Addgene	Cat# 52963; RRID: Addgene_52963

**Chemicals, peptides and recombinant proteins**		

Agarose (standard grade)	Euroclone	Cat# EMR010500
Agarose Low EEO (for 2D-gels and alkaline gels)	US Biological	Cat# A1015
Agarose, low melting point (for plug casting)	Lonza	Cat# 50104
Ampicillin	Sigma-Aldrich	Cat# A9518
Boric acid	VWR	Cat# 20185
Bromocresol green	Sigma-Aldrich	Cat # 114359
Bromophenol blue	Sigma-Aldrich	Cat# B0126
BSA (molecular biology grade)	NEB	Cat# B9000
BsmBI-v2	NEB	Cat# R0739S
CaCl_2_	Sigma-Aldrich	Cat# C1016
Chloroform (molecular biology grade)	Sigma-Aldrich	Cat# C2432
CutSmart Buffer (10x)	NEB	Cat# B6004S
Dimethyl sulfoxide (DMSO), sterile	Sigma-Aldrich	Cat# D2438
DNase I (RNase-free, lyophilized)	Worthington	Cat# LS006333
dNTP Set (100 mM solutions)	Invitrogen	Cat# 10297018
Doxycycline hydrochloride	Sigma-Aldrich	Cat# D9891; CAS: 24390-14-5
DpnI restriction enzyme	NEB	Cat# R0176L
DTT (dithiothreitol), 1 M	Sigma-Aldrich	Cat# D9779
EDTA	Sigma-Aldrich	Cat# E9884
EGTA	Sigma-Aldrich	Cat# E3889
Ethanol, absolute (molecular biology grade)	Sigma-Aldrich	CAS: 64-17-5
Ethidium Bromide solution (10 mg/mL)	Sigma-Aldrich	Cat# E1510
Ficoll 400	Sigma-Aldrich	Cat# F4375
Geneticin G418 sulfate (powder)	Life Technologies	Cat# 11811031
Glycerol (molecular biology grade)	Sigma-Aldrich	Cat# G5516
HCl, 37% (12 N)	Sigma-Aldrich	Cat# 320331
HinfI	NEB	Cat# R0155L
Isopropanol (molecular biology grade)	Sigma-Aldrich	CAS: 67-63-0
KCl	Sigma-Aldrich	Cat# P9333
Klenow Fragment (3'→5′ exo-)	NEB	Cat# M0212S
L-Glutamine, 200 mM	Euroclone	Cat# LOBE1760F
LB Broth, Miller	BD Diagnostic Systems	Cat# 244620
Magnesium acetate	Sigma-Aldrich	Cat# M5661
MgCl_2_	Sigma-Aldrich	Cat# M8266
MspI	NEB	Cat# R0106L
N-Lauroylsarcosine sodium salt	Sigma-Aldrich	Cat# L9150
NaOH	Sigma-Aldrich	Cat# 1.09137
Phenol:chloroform:isoamyl alcohol (25:24:1)	Sigma-Aldrich	Cat# P2069
PMSF (phenylmethylsulfonyl fluoride)	Sigma-Aldrich	Cat# 78830
Polybrene (Hexadimethrine bromide)	Sigma-Aldrich	Cat# H9268
Polyethylenimine Max 40000 (PEI Max)	Histo-Line Laboratories	Cat# 24765-1
Potassium phosphate monobasic	Sigma-Aldrich	Cat# P0662
Protease Inhibitor Cocktail (cOmplete, EDTA-free)	Roche	Cat# 11836170001
Proteinase K (20 mg/mL stock solution)	Roche	Cat# 3115887001
Puromycin dihydrochloride	Adipogen	Cat# AG-CN2-0078-M100
Q5 High-Fidelity DNA Polymerase	NEB	Cat# M0491S
Quick Ligation Kit	NEB	Cat# M2200S
RNase A (10 mg/mL, DNase-free)	Sigma-Aldrich	Cat# R5000
RsaI	NEB	Cat# R0167L
SDS 20% (w/v) solution	ITW Reagents	Cat# A3942,1000
Skim milk powder	Millipore	Cat# 70166
Sodium acetate, 3 M, pH 5.2	Sigma-Aldrich	CAS: 127-09-3
Sodium citrate dihydrate	Sigma-Aldrich	Cat# W302600
Sodium deoxycholate	Sigma-Aldrich	Cat# D6750
Sodium phosphate dibasic	Sigma-Aldrich	Cat# S3264
Sodium chloride	Carlo Erba	Cat# 479687
Sucrose	Sigma-Aldrich	Cat# S0389
T4 DNA Ligase (400,000 U/mL)	NEB	Cat# M0202S
T4 Polynucleotide Kinase (PNK)	NEB	Cat# M0201S
Tris base	Sigma-Aldrich	Cat# T1503
Triton X-100 (10% solution, cell culture tested)	Sigma-Aldrich	Cat# X100
Trypsin-EDTA	Euroclone	Cat# ECB3052D
Tween-20	Sigma-Aldrich	Cat# P9416
Xylene cyanol	Sigma-Aldrich	Cat# X4126
α-32P-dCTP (3000 Ci/mmol, 10 mCi/mL)	PerkinElmer	Cat# NEG513Z
γ-32P-ATP (6000 Ci/mmol, 10 mCi/mL)	PerkinElmer	Cat# NEG5502Z

**Critical commercial assays**		

GenePrint 10 System (STR cell line authentication)	Promega	Cat# B9510
MycoAlert Mycoplasma Detection Kit	Lonza	Cat# LT07-418
NucleoBond Xtra Maxi Kit (maxiprep)	Macherey-Nagel	Cat# 740414.50
Prime-a-Gene Labeling System (random-prime labeling)	Promega	Cat# U1100
QuBit dsDNA Assay Kit, Broad Range	Invitrogen	Cat# Q32850
SuperSignal West Pico Plus Chemiluminescent Substrate	Thermo Scientific	Cat# 34580
Wizard Plus SV Miniprep DNA Purification System	Promega	Cat# A1330
Wizard SV Gel and PCR Clean-Up System	Promega	Cat# A9281

**Deposited data**		

Original images and source data (Zanella et al., 2026)^1^	Zanella et al.^1^	Mendeley Data: https://doi.org/10.17632/hwf38mddyt.1

**Experimental models: cell lines**		

Human: HT1080 (fibrosarcoma, male)	ATCC	Cat# CCL-121; RRID: CVCL_0317
Human: HEK293-T (embryonic kidney, female)	ATCC	Cat# CRL-3216; RRID: CVCL_0063
Human: HT1080 Lenti-iCas9^D10A^-Neo (generated in this study)	Zanella et al.^1^; Doksani Lab plasmid #123	N/A
Human: HT1080 Lenti-iCas9^H840A^-Neo (generated in this study)	Zanella et al.^1^; Doksani Lab plasmid #124	N/A

**Oligonucleotides**		

Telomere-specific sgRNA-FW: 5′-CACCGTTAGGGTTAGGGTTAGGGTTA-3′	Zanella et al.^1^	N/A
Telomere-specific sgRNA-RV: 5′-AAACAACCCTAACCCTAACCCTAAC-3′	Zanella et al.^1^	N/A
Alpha satellite-specific sgRNA-FW: 5′-CACCGGAATCTGCAAGTGGATATTG-3′	Zanella et al.; Gao et al.^1^^,^^7^	N/A
Alpha satellite-specific sgRNA-RV: 5′- AAACCAATATCCACTTGCAGATTCC-3′	Zanella et al.; Gao et al.^1^^,^^7^	N/A
C-strand telomeric probe: (CCCTAA)_4_	Zanella et al.^1^	N/A
G-strand telomeric probe: (TTAGGG)_5_	Zanella et al.^1^	N/A
Alpha satellite primer α27: 5′-CATCACAAAGAAGTTTCTGAGAATGCTTC-3′	Choo et al.; Catacchio et al.^10^^,^^11^	N/A
Alpha satellite primer α30: 5′-TGCATTCAACTCACAGAGTTGAACCTTCC-3′	Choo et al.; Catacchio et al.^10^^,^^11^	N/A
Cas9^D10A^ mutagenic FW: 5′-cctggCcatcggcaccaactctgtgg-3′	Zanella et al.^1^	N/A
Cas9^D10A^ mutagenic RV: 5′-cgatgGccaggccgatgctgtacttc-3′	Zanella et al.^1^	N/A
Cas9^H840A^ mutagenic FW: 5′-gtggacGCtatcgtgcctcagagctttc-3′	Zanella et al.^1^	N/A
Cas9^H840A^ mutagenic RV: 5′-cacgataGCgtccacatcgtagtcggac-3′	Zanella et al.^1^	N/A

**Recombinant DNA**		

Lenti-iCas9-Neo (doxycycline-inducible FLAG-Cas9 backbone)	Addgene	Cat# 85400; RRID: Addgene_85400
Lenti-iCas9^D10A^-Neo	Zanella et al.^1^; Doksani Lab plasmid #123	N/A
Lenti-iCas9^H840A^-Neo	Zanella et al.^1^; Doksani Lab plasmid #124	N/A
LentiGuide-Puro (sgRNA expression backbone)	Addgene	Cat# 52963; RRID: Addgene_52963
Teloguide: LentiGuide-Puro + telomere sgRNA	Zanella et al.^1^; Doksani Lab plasmid #129	N/A
Alphaguide: LentiGuide-Puro + alpha satellite sgRNA	Zanella et al.^1^; Doksani Lab plasmid #138	N/A
pSty11 (800 bp EcoRI telomeric probe template)	Titia de Lange Lab/Addgene	Cat# 12401; RRID: Addgene_12401
pMD2.G - lentiviral packaging 1/3	Addgene	Cat# 12259
pMDLg/pRRE - lentiviral packaging 2/3	Addgene	Cat# 12251
pRSV-Rev - lentiviral packaging 3/3	Addgene	Cat# 12253
α-satellite pSP73 (316 bp probe template, this study)	Zanella et al.^1^; Doksani Lab plasmid #139	N/A

**Software and algorithms**		

ImageJ/Fiji v1.54 (gel quantification)	Schneider et al.^12^	https://imagej.nih.gov/ij/; RRID: SCR_002285
GraphPad Prism v9+	GraphPad Software	https://www.graphpad.com/; RRID: SCR_002798
Typhoon scanner control software	Cytiva/GE Healthcare	N/A

**Other**		

Amersham Typhoon 5 PhosphorImager scanner	Cytiva	RRID: SCR_025702
ChemiDoc Imaging System (or equivalent gel documentation system)	Bio-Rad	N/A
DMEM High Glucose (cell culture medium)	Euroclone	Cat# ECB7501L
DNA ladder, 1 kb, dye-free (alkaline gels)	NEB	Cat# N3232L
Lambda DNA-HindIII digest	NEB	Cat# N3012L
Gel electrophoresis apparatus with recirculating buffer pump (2D-gels)	Thermo Scientific	Cat# A5
Hybond-N^+^ nylon membrane	Cytiva	Cat# RPN203B
Hybridization cassette	VWR	Cat# RPN11645
Hybridization Mesh Sheets	Thermo Scientific	Cat# 222053
MicroSpin G-25 Columns (purification of end-labeled probes)	GE Healthcare	Cat# 27532501
MicroSpin G-50 Columns (purification of randomly primed probes)	GE Healthcare	Cat# 27533001
Protran nitrocellulose membrane, 0.45 μm	GE Healthcare	Cat# 10600002
Penicillin/Streptomycin (100× stock)	Euroclone	Cat# ECB3001L
Peristaltic pump (for alkaline gel electrophoresis)	Variable — any model rated 5–20 mL/min	N/A
Phosphor storage screen (BAS-MS type)	GE Healthcare	Cat# 28-9564-76
Plug molds (0.5 mL CHEF plug molds)	Bio-Rad	Cat# 1703713
QuBit 4 Fluorometer	Invitrogen	Cat# Q33226
Safe Imager 2.0 Blue-Light Transilluminator	Thermo Fisher Scientific	Cat# G6600
Syringe filters, 0.45 μm, low protein binding (PVDF)	Merck Millipore	Cat# SLHV033RS
Standard horizontal gel electrophoresis apparatus	Thermo Scientific	Cat# A2-BP
Tet-System Approved FBS (tetracycline-free fetal bovine serum)	Euroclone	Cat# ECS0182L
UV Crosslinker Stratalinker 1800 (254 nm and 365 nm bulbs)	Stratagene	Cat# 400072
Whatman 3MM chromatography paper	Cytiva	Cat# 3030-917
Wide-bore pipette tips, 1000 μL	Corning	Cat# T-1005-WB-C

**CRITICAL:** Use tetracycline-free FBS, low-EEO agarose and freshly prepared alkaline electrophoresis buffer. Do not replace tetracycline-free FBS with conventional FBS, which may contain trace tetracyclines and cause basal Cas9 expression in uninduced controls. Low quality agarose may not perform as well in separating DNA structures in the second dimension, while stored alkaline electrophoresis buffer may not maintain the pH required for complete DNA denaturation. Other reagents and instruments may be replaced with equivalent products, provided their performance is validated for the relevant step. Alternatives tested in our laboratory are indicated in the corresponding steps.

## Materials and equipment

Prepare all buffers with molecular-biology-grade water (18.2 MΩ·cm). Filter-sterilize (0.22 μm) where indicated. Adjust pH at 20–25°C unless stated otherwise.

**Table:** TNE Buffer (for genomic DNA extraction)

Reagent	Stock	Final conc.	Volume/Amount
Tris-HCl, pH 8.0	1 M	10 mM	10 mL
NaCl	5 M	100 mM	20 mL
EDTA, pH 8.0	0.5 M	10 mM	20 mL
Molecular biology-grade H_2_O	—	—	950 mL
Total			1 L

Prepare in advance. Store at 20–25°C for up to 6 months.

**Table:** TNES Lysis Buffer (TNE + SDS)

Reagent	Stock	Final conc.	Volume/Amount
Tris-HCl, pH 8.0	1 M	10 mM	10 mL
NaCl	5 M	100 mM	20 mL
EDTA, pH 8.0	0.5 M	10 mM	20 mL
SDS	20% (w/v)	1% (w/v)	50 mL
Molecular biology-grade H_2_O	—	—	900 mL
Total			1 L

Pre-warm to 37°C before use to dissolve any SDS precipitation.

**Table:** TES Buffer

Reagent	Stock	Final conc.	Volume/Amount
Tris-HCl, pH 7.4	1 M	10 mM	10 mL
EDTA, pH 8.0	0.5 M	10 mM	20 mL
SDS	20% (w/v)	0.1% (w/v)	5 mL
Molecular biology-grade H_2_O	—	—	965 mL
Total			1 L

Pre-warm to 37°C before use to dissolve any SDS precipitation.

**Table:** 1× PBS

Reagent	Stock	Final conc.	Volume/Amount
NaCl	powder, 58.44 g/mol	137 mM	8 g
KCl	powder, 74.55 g/mol	2.68 mM	0.2 g
Sodium phosphate dibasic	powder, 141.96 g/mol	8 mM	1.14 g
Potassium phosphate monobasic	powder, 136.09 g/mol	1.47 mM	0.2 g
Adjust to pH 6.79 with HCl	—	—	to 1 L
Total			1 L

Store at 20–25°C up to 6 months.

**Table:** Hypotonic Buffer (for nuclei isolation in DNase I alternative)

Reagent	Stock	Final conc.	Volume/Amount
Tris-HCl, pH 7.4	1 M	10 mM	1 mL
MgCl_2_	1 M	1.5 mM	150 μL
KCl	1 M	10 mM	1 mL
Sucrose (molecular biology grade)	powder, 342.30 g/mol	340 mM	11.64 g
Protease inhibitor cocktail (50× tablet dissolved in x 10mL of H_2_O)	50×	1×	2 mL
Molecular biology-grade H_2_O	—	—	to 100 mL
Total			100 mL

Prepare on the day of use. Supplement with protease inhibitor cocktail (1× final) immediately before use. Keep on ice at all times.

**Table:** Nuclei Wash Buffer (NWB)

Reagent	Stock	Final conc.	Volume/Amount
Tris-HCl, pH 7.4	1 M	10 mM	1 mL
KCl	1 M	60 mM	6 mL
NaCl	5 M	15 mM	300 μL
MgCl_2_	1 M	5 mM	500 μL
Sucrose	powder, 342.30 g/mol	300 mM	10.27 g
Molecular biology-grade H_2_O	—	—	to 100 mL
Total			100 mL

Prepare fresh. Store at 4°C.

**Table:** DNase I Reaction Buffer

Reagent	Stock	Final conc.	Volume/Amount
NWB (see above)	1×	1×	200 μl per sample
CaCl_2_	100 mM	2 mM	2 μL per 100 μL reaction
BSA (molecular biology grade)	10 mg/mL	0.1 mg/mL	1 μL per 100 μL reaction
Total			200 μl per sample

Prepare fresh, right before use, by supplementing NWB with CaCl_2_ and BSA, as indicated below. The exact volume depends on the number of samples; a typical experiment requires 200 μL per sample.

**Table:** DNase I Stop Buffer

Reagent	Stock	Final conc.	Volume/Amount
EDTA, pH 8.0	0.5 M	50 mM	10 mL
EGTA, pH 8.0	0.5 M	10 mM	2 mL
Molecular biology-grade H_2_O	—	—	88 mL
Total			100 mL

Prepare in advance. Store at 20–25°C.

**Table:** 5× TBE Running Buffer (for 1D and 2D agarose gels)

Reagent	Stock	Final conc.	Volume/Amount
Tris base	powder 121.14 g/mol	445 mM	53.9 g
Boric acid	powder 61.83 g/mol	445 mM	27.5 g
EDTA, pH 8.0	0.5 M	10 mM	20 mL
Molecular biology-grade H_2_O	—	—	to 1 L
Total			1 L

Prepare a 5× TBE stock and dilute 1:10 before use. Store at 20–25°C; discard if precipitate forms. Use 0.5× TBE for both the first- and second-dimension gels.

**Table:** 10× Alkaline Electrophoresis Buffer

Reagent	Stock	Final conc.	Volume/Amount
NaOH, 10 M	10 M	0.5 M	50 mL
EDTA, pH 8.0	0.5 M	0.01 M	20 mL
Molecular biology-grade H_2_O	—	—	930 mL
Total			1 L

Prepare fresh monthly. Use within 4 weeks, at 1× final concentration unless stated otherwise. Wear PPE when handling concentrated NaOH.

**Table:** 6× Alkaline Gel-Loading Buffer

Reagent	Stock	Final conc.	Volume/Amount
NaOH, 10 M	10 M	0.3 M	0.3 mL
EDTA, pH 8.0	500 mM	6 mM	0.12 mL
Ficoll type 400	powder	18% w/v	1.8 g
Bromocresol green	powder	0.15% w/v	0.015 g
Xylene cyanol	powder	0.25% w/v	0.025 g
Molecular biology-grade H_2_O	—	—	to 10 mL
Total			10 mL

Store at 4°C for up to a year. Equilibrate at 20–25°C and mix well before use.

**Table:** Neutralizing Solution (for alkaline gel and Southern blot)

Reagent	Stock	Final conc.	Volume/Amount
NaCl	powder 58.44 g/mol	1.5 M	87.66 g
Tris-HCl, pH 7.0	1 M	0.5 M	500 mL
Molecular biology-grade H_2_O	—	—	to 1 L
Total			1 L

Store at 20–25°C for up to 3 months.

**Table:** Depurination Solution (for Southern blot)

Reagent	Stock	Final conc.	Volume/Amount
HCl	37% (12 N)	0.25 N	21 mL
Molecular biology-grade H_2_O	—	—	to 1 L
Total			1 L

Prepare fresh or store at 20–25°C up to 1 month.

**Table:** Denaturing Solution (for Southern blot)

Reagent	Stock	Final conc.	Volume/Amount
NaCl	powder 58.44 g/mol	1.5 M	87.66 g
NaOH (pellets)	—	0.5 M	20 g
Molecular biology-grade H_2_O	—	—	to 1 L
Total			1 L

Prepare fresh or store at 20–25°C for up to 1 month.

**Table:** 20× SSC

Reagent	Stock	Final conc.	Volume/Amount
NaCl	powder 58.44 g/mol	3 M	175.32 g
Sodium citrate dihydrate	powder 294.10 g/mol	0.3 M	88.2 g
Adjust pH to 7.0 with concentrated HCl	—	—	—
Molecular biology-grade H_2_O	—	—	to 1 L
Total			1 L

Autoclave or filter-sterilize (0.22 μm), store at 20–25°C. Dilute 1:2 to obtain 10× SSC: Southern blot transfer buffer.

**Table:** Membrane stripping solution (0.1% SDS, 0.1× SSC)

Reagent	Stock	Final conc.	Volume/Amount
SDS	20% (w/v)	0.1% (w/v)	2.5 mL
SSC	20×	0.1×	2.5 mL
Molecular biology-grade H_2_O	—	—	495 mL
Total			500 mL

Prepare fresh on the day of use; use hot. Do not store.

**Table:** Church Hybridization Buffer (Church mix)

Reagent	Stock	Final conc.	Volume/Amount
Sodium phosphate, pH 7.2	1 M	0.5 M	250 mL
EDTA, pH 8.0	0.5 M	1 mM	1 mL
SDS, 20% w/v	20%	7% (w/v)	175 mL
BSA (bovine serum albumin, powder)	powder	1% (w/v)	5 g
Molecular biology-grade H_2_O	—	—	to 500 mL
Total			500 mL

Prepare as a 500-mL stock. Heat to 65°C, while stirring, to dissolve SDS fully. Store at 20–25°C; stable for 6 months. Incubate at 55–65°C for 10–15 min before use. Shake well and check for precipitates, before use.

**Table:** Church Wash Buffer

Reagent	Stock	Final conc.	Volume/Amount
Sodium phosphate, pH 7.2	1 M	40 mM	40 mL
EDTA, pH 8.0	0.5 M	1 mM	2 mL
SDS,20% (w/v)	20%	1% (w/v)	50 mL
Molecular biology-grade H_2_O	—	—	to 1000 mL
Total			1000 mL

Prepare as a 1-L stock. Pass through a 0.45 μm filter unit. Store at 20–25°C. Prewarm at 65°C or at the washing temperature before use.

**Table:** 2× Laemmli SDS-PAGE Loading Buffer (for western blot)

Reagent	Stock	Final conc.	Volume/Amount
Tris-HCl, pH 6.8	1 M	120 mM	600 μL
Glycerol (100%)	100%	20% (v/v)	1 mL
SDS, 20% w/v	20%	4% (w/v)	1 mL
Bromophenol blue, 0.5% (w/v)	0.5%	0.02%	200 μL
Molecular biology-grade H_2_O	—	—	to 4 mL
Total			4 mL

Prepare 4-mL aliquots without DTT. Store at −20°C for up to 1 year. Add 1 mL of DTT 1M (200 mM final) on the day of use.

**Table:** 1× Proteinase K Buffer for agarose plug

Reagent	Stock	Final conc.	Volume/Amount
Tris-HCl, pH 8.0	1M	10 mM	1 mL
EDTA, pH 8.0	0.5 M	100 mM	20 mL
Sodium deoxycholate	powder	0.2% (w/v)	200 mg
N-Lauryl sarcosine sodium salt	powder	1% (w/v)	1 g
Molecular biology-grade H_2_O	—	—	to 100 mL
Total			100 mL

Prepare in advance and store at 20–25°C up to 1 month.

**Table:** 10× OLB Buffer

Reagent	Stock	Final conc.	Volume/Amount
Tris-HCl, pH 6.8	1 M	500 mM	500 μL
Magnesium acetate	1 M	100 mM	100 μL
DTT (add fresh on day of use)	1 M	1 mM	1 μL
BSA (molecular biology grade)	10 mg/mL	0.5 mg/mL	50 μL
dATP	100 mM	0.6 mM	6 μL
dGTP	100 mM	0.6 mM	6 μL
dTTP	100 mM	0.6 mM	6 μL
Molecular biology-grade H_2_O	—	—	331 μL
Total			1 mL

Prepare fresh or store at −20°C for up to 6 months.

## Step-by-step method details

### Generation of doxycycline-inducible Cas9 nickase cell lines

**Timing: 10–14 days**

This section describes the generation of stable HT1080 cell pools co-expressing a doxycycline-inducible FLAG-tagged Cas9 nickase (either D10A or H840A) and a constitutively expressed repeat-targeting sgRNA. Each nickase variant is generated separately and parallel analysis allows comparison of strand-specific targeting of telomeric repeats in downstream analysis. The two components are delivered in two sequential transductions, each followed by antibiotic selection, so that the nickase and the guide are under independent control: the nickase is silent until doxycycline is added, whereas the sgRNA is expressed constitutively and is therefore already present when the nickase is expressed. The output of this section is a dual-selected, heterogeneous cell pool that is the input for doxycycline induction (Step 1 of the graphical abstract, 10–14 days). The essential checkpoint is the anti-FLAG western blot in step 6: do not proceed to the second transduction until doxycycline-dependent nickase expression has been confirmed.

1. Expand HT1080 cells in DMEM supplemented with 10% Tet-free FBS and 2 mM L-glutamine.
  a. Seed the verified iCas9-Neo pool 24 h in advance so that it is at 40%–50% confluency on the day of transduction.

***Note:*** We use 15-cm dishes at this stage in order to have sufficient material for the parallel telomere and alpha satellite conditions.

2. Replace culture medium with 10 mL of the filtered lentiviral supernatant supplemented with polybrene at 8 μg/mL final concentration.

**CRITICAL:** Set up parallel transductions for Cas9^D10A^ and Cas9^H840A^ in separate dishes from the same HT1080 passage. Never mix the two nickase constructs in a single dish; the resulting cell population would generate nicks on both strands simultaneously, making strand-specific alkaline gel analysis difficult to interpret.

3. Incubate for 48 h at 37°C in 5% CO_2_ without changing the medium.
4. Replace the viral supernatant with fresh complete DMEM containing 10% Tet-free FBS and 2 mM L-glutamine. Culture for a further 24 h to allow recovery before selection.
5. Begin G418 (neomycin) selection.
  a. Replace medium with DMEM supplemented with 10% Tet-free FBS, 2 mM L-glutamine and 400 μg/mL G418.
  b. Maintain an untransduced HT1080 dish under the same selection conditions as a killing control.
  c. Replace selection medium every 2–3 days for 7–10 days until all cells in the killing control dish are dead.

***Note:*** The effective G418 killing concentration should be titrated for each cell line used. For HT1080, 400 μg/mL gives complete killing of uninfected cells within 5–7 days. The maintenance concentration after pool selection is 200 μg/mL.

***Note:*** A 6 cm plate seeded at the same density and carried through the identical selection schedule is used as a kill control.

6. Expand surviving iCas9-Neo pools in DMEM supplemented with 10% Tet-free FBS, 2 mM L-glutamine and 200 μg/mL G418 (maintenance). Verify Cas9 nickase expression by western blot before proceeding to sgRNA transduction.
  a. Seed 5 × 10^5^ iCas9-Neo cells in a 6-cm dish. Add doxycycline to 0.5 μM (from a 10 mM stock in ddH_2_O stored at −20°C). Culture for 16 h.
  b. Harvest cells, lyse in Laemmli buffer 2× (with 200 mM DTT added fresh) at 1 × 10^7^ cells/mL. Boil 4 min at 95°C. Shear genomic DNA in the protein lysate by passing it 4 times through a 28-gauge insulin syringe needle.
  c. Load the equivalent of 1.2 × 10^5^ cells per lane on an 8% SDS-PAGE gel. Run at 110 V for 90 min. Transfer 12–16 h at 150 mA at 4°C onto a 0.45-μm nitrocellulose membrane.
  d. Block with 5% skim milk in PBS-T (PBS + 0.1% Tween-20) for 1 h at 20–25°C. Incubate with anti-FLAG antibody (1:1,000 in 5% milk/PBS-T) for 12–16 h at 4°C.
  e. Wash 3 times for 5 min each in PBS-T. Incubate with HRP-linked anti-mouse IgG secondary antibody (1:5,000) for 30–45 min at 20–25°C. Detect with SuperSignal West Pico Plus.

***Note:*** The expected result is a clear band at ∼160 kDa in the doxycycline-induced lane, absent in the uninduced control; ∼37 kDa GAPDH serves as the loading control.

***Note:*** Any abundant housekeeping protein whose molecular weight is well separated from ∼160 kDa is suitable as loading control; we have used GAPDH (∼37 kDa) and α-tubulin (∼50 kDa) interchangeably.

TROUBLESHOOTING: see problem 1 for weak or uneven Cas9 signal.

**Pause point:** G418-selected iCas9-Neo pools (before sgRNA transduction) can be cryopreserved in 90% FBS + 10% DMSO and stored in liquid nitrogen. After freezing, one vial of the batch is tested in a mock experiment, where the cells are thawed, expanded and induced with doxycycline and Cas9 expression is verified by western blot. This quality control is performed once per batch. Using a well-tested, cryopreserved pool at this stage prevents the need to re-derive cell lines if later experiments fail.

**CRITICAL:** Cells should not be kept in culture for more than two weeks or subjected to repeated freeze-thaw cycles, as we have observed that this leads to reduced nicking efficiency in alkaline gels. Freeze the cells in large batches, right after testing for Cas9 induction by western blot.

7. Transduce the verified iCas9-Neo cells (at 40%–50% confluency in 15-cm dishes) with the sgRNA lentiviral supernatant.
  a. Seed the verified iCas9-Neo pool 24 h in advance so that it is at 40%–50% confluency on the day of transduction. We use 15-cm dishes at this stage in order to have sufficient material for the parallel telomere and alpha satellite conditions.
  b. Replace the medium with 15 mL of filtered sgRNA lentiviral supernatant per 15-cm dish, supplemented with polybrene at 8 μg/mL final concentration. This corresponds to the same supernatant-to-surface-area ratio used in step 2.
  c. Incubate for 48 h at 37°C in 5% CO_2_ without changing the medium.
    **CRITICAL:** In our hands transducing with the undiluted supernatant allows for sufficient sgRNA expression in the cell population to detect nick formation in alkaline gels, without affecting cell viability. Viral titers may vary across different labs, due to cell culture reagents and other conditions, therefore we advise to carefully monitor cell viability in control infections in initial experiments and if necessary, dilute the viral supernatant used for infection, as described in step 10 of “Lentivirus production in HEK293-T cells”.
  d. To detect i-loops at telomeric or alpha satellite repeats, transduce with the generated Teloguide or Alphaguide, respectively.
  e. Locus specificity can be assessed by generating separate dishes for each sgRNA; they will serve as the non-targeted repeat controls for each other.
8. Begin puromycin selection 48 h post-sgRNA transduction.
  a. Replace medium with DMEM supplemented with 10% Tet-free FBS, 2 mM L-glutamine and 2 μg/mL puromycin.
  b. Select cells for 3–5 days until the untransduced killing control is completely dead. Similarly to the G418 step, a 6 cm plate of untransduced cells, seeded at matched density and carried through the identical schedule, is a sufficient killing control once the puromycin concentration has been established for the cell line.

***Note:*** The effective puromycin killing concentration should be titrated for each cell line used.

9. Expand double-transgenic cell pools (iCas9 nickase + sgRNA) in DMEM supplemented with 10% Tet-free FBS, 2 mM L-glutamine, 200 μg/mL G418 and 1 μg/mL puromycin (maintenance). The cells are ready for doxycycline induction experiments at this point as heterogeneous pools.

***Note:*** In this protocol downstream experiments are performed using the heterogeneous pool of cells, after antibiotic selection. This minimizes potential clonal effects on repeat length, genetic background or basal DNA damage levels.

***Note:*** HT1080 cells tolerate continuous dual-antibiotic selection, therefore we maintain it throughout the experiment. For cell lines that do not tolerate it as well, only the two-days puromycin selection of the sgRNA transduction can be applied. In this case, a western blot of Cas9 induction is a crucial control, not to be omitted.

***Note:*** The two-component lentiviral design used here requires two sequential transductions and two rounds of antibiotic selection and is therefore best suited to immortalized lines that tolerate these steps. For primary cells, iPSCs or other models that do not tolerate sequential transductions and selection, purified Cas9^D10A^ or Cas9^H840A^ protein pre-complexed with the repeat-targeting sgRNA can be delivered as a ribonucleoprotein (RNP) by nucleofection or electroporation.^13^ In that case, the experimental conditions, e.g. starting amount of cells, harvest timing and nicking efficiency would need to be optimized ad hoc.

### Doxycycline induction of targeted SSBs and cell harvest

**Timing: ∼18 h (1 h plating check + 16 h induction + ∼1 h harvest)**

Here we describe the induction of Cas9 nickase expression and the harvest of the induced cells. Adding doxycycline activates the Tet-On promoter and allows the nickase, guided by the constitutively expressed sgRNA, to introduce single-strand breaks at the targeted repeat. The output is two matched sample from every dish: an agarose-plug aliquot used to verify nicking on an alkaline gel, and a bulk cell pellet used for high-molecular-weight DNA extraction and 2D-gel analysis (Step 2 of the graphical abstract, 16 h). The 16 h induction window has been empirically determined to allow robust i-loop accumulation while limiting secondary DNA damage responses. The uninduced control is the most important control and should always be run in parallel from the same cell pool on the same day.

10. Plate double-transgenic HT1080 cells in 15-cm dishes 24 h before the experiment. Aim for ∼70% confluency at the time of doxycycline addition. For a standard 2D-gel analysis, use one dish per condition (induced and uninduced) to obtain at least 20 μg of purified genomic DNA per condition.
11. Add doxycycline hydrochloride directly to the culture medium at a final concentration of 0.5 μM using a fresh or single-use aliquot of a 10 mM stock prepared in ddH_2_O, filter-sterilized and stored at −20°C, protected from light. For uninduced controls, add an equivalent volume of the doxycycline vehicle, ddH_2_O.

**CRITICAL:** Use a doxycycline stock that has not undergone more than three freeze-thaw cycles and is no older than 6 months from preparation. Doxycycline is sensitive to repeated freeze-thaw cycles or light exposure. A degraded stock can result in inefficient Cas9 nickase expression; a common cause of experiment failure, failing to induce SSBs and an i-loop arc in 2D-gels.

12. Incubate for 16 h at 37°C in 5% CO_2_. Prepare all material and reagents for rapid cell harvest at the end of the incubation to minimize post-induction nick repair.
13. At the end of the 16-h incubation, rapidly aspirate the medium from each dish, add 10 mL of PBS, pre-warmed to 37°C, rock gently for 10 s and aspirate the PBS wash.
14. Harvest the cells by trypsinization.
  a. Add 2 mL of 0.25% trypsin-EDTA per 15-cm dish and incubate for 2–3 min at 37°C until cells detach.
  b. Neutralize with 5 mL complete DMEM supplemented with 10% FBS and 2 mM L-glutamine, collect the cells and transfer to a 15-mL conical tube.
  c. Count the harvested cells by your preferred method and pellet the cells at 300 × g for 5 min at 4°C.
  d. Aspirate supernatant completely and wash with 10 mL of ice-cold PBS.
15. From each condition, reserve 3–4 x 10^6^ cells (1 × 10^6^ cells per plug) in a separate 1.5-mL microcentrifuge tube for agarose plug preparation and alkaline gel electrophoresis ((“Verification of nick formation by alkaline agarose gel electrophoresis”, steps 36–44). Pellet this aliquot at 300 × g for 5 min at 4°C and keep on ice.
  a. Save the remaining cells (typically 5–16 × 10^6^ per dish) for genomic DNA extraction ((“Extraction of high-molecular-weight genomic DNA”, steps 26–35).
  b. If multiple 15-cm dishes are used per condition, pool the trypsinized cells before the final centrifugation step.

***Note:*** Set aside agarose plug aliquots before any further processing. Embedding cells in agarose minimizes mechanical shear, preserving intact genomic DNA for a more accurate analysis of SSBs in alkaline gel electrophoresis.

### DNase I treatment of isolated nuclei for nick induction (Cas9-independent control method)

**Timing: 1 day**

At low concentrations, DNase I generates SSBs and short gaps in chromatinized DNA, providing a Cas9- and sgRNA-independent source of single-stranded DNA damage (Step 2 of the graphical abstract).^14^ Limited DNase I treatment of isolated nuclei induces i-loop formation at repeats and therefore provides a positive control for the downstream 2D-gel and Southern blotting workflow.^15^ This method may also be applied directly to purified genomic DNA as an in vitro damage control, described at the end of this section. The purpose of this section is therefore to generate a positive control in which i-loops are known to form, so that a negative 2D-gel result in the Cas9-induced samples can be attributed to the induction rather than to a failure of the downstream detection workflow. Its output is a suspension of nuclei (carrying damaged DNA) that enters the DNA extraction workflow at step 26. This section can be run independently of the lentiviral engineering described above, in any cell type.

16. Harvest cells by trypsinization as in Step 14 of “Doxycycline induction of targeted SSBs and cell harvest”. A standard preparation requires 40 × 10^6^ cells per condition.
  a. Wash the cell pellet once with 5 mL of ice-cold PBS.
  b. Centrifuge cells at 300 × g for 5 min at 4°C.
  c. Aspirate the supernatant.

**CRITICAL:** All steps from this point until DNase I treatment must be performed at 4°C or on ice to prevent premature nuclease activation and maintain nuclei integrity.

17. Gently resuspend the cell pellet in 5 mL ice-cold hypotonic buffer (freshly supplemented with protease inhibitor cocktail). Centrifuge at 330 × g for 5 min at 4°C.
18. Remove the supernatant and resuspend the pellet again in 10 mL of ice-cold hypotonic buffer. Incubate on ice for 10 min.

***Note:*** This hypotonic incubation causes cells to swell, facilitating membrane disruption in the next step.

19. Add Triton X-100 to 0.33% (v/v) final concentration by adding 1/30 volume of a 10% Triton X-100 stock. Close the tube and invert exactly twice. Do not vortex. Incubate on ice for 5 min.

**CRITICAL:** Excess Triton X-100 or prolonged detergent incubation might compromise nuclei integrity and release genomic DNA causing nuclei to aggregate. Invert the tube exactly twice and keep strictly to the 5-min incubation time.

20. Pellet nuclei at 1000 × g for 5 min at 4°C. Aspirate the supernatant, which contains cytoplasmic contents and detergent.

***Note:*** Inspect a small aliquot by phase-contrast microscopy to confirm nuclei integrity and absence of intact cells. Good preparations should contain intact nuclei without the bright cytoplasmic halo seen around the nucleus in intact cells.

21. Wash nuclei twice with 10 mL ice-cold NWB. For each wash, resuspend by gentle inversion five times, then centrifuge at 1000 × g for 5 min at 4°C.
22. Resuspend the final nuclei pellet in ice-cold NWB at 25 × 10^6^ nuclei/mL. You will need 200 μL of this suspension per treatment condition. Keep on ice and proceed immediately to DNase I treatment.
23. Set up DNase I treatment reactions in microcentrifuge tubes with one volume (200 μL) of DNase I reaction buffer. Prepare at least two conditions in parallel: 0 and 13 ng/μL DNase I in the reaction buffer, which will be diluted to 0 and 6.5 ng/μL, respectively, after addition of an equal volume of nuclei suspension. Keep the tubes in a thermomixer at 25°C, while preparing to add the nuclei suspension.
  ***Note:*** DNase I concentration should be empirically optimized for each stock. The final concentration of 6.5 ng/μL is indicative and specific to our reaction conditions. However, DNase I activity can vary between lots, reconstituted stocks and storage conditions. When using a new stock, first test a small concentration range, for example 2.5, 5 and 10 ng/μL final DNase I and choose the lowest concentration that produces detectable nicking while preserving most DNA at high-molecular-weight (HMW). Once a stock is prepared, make intermediate dilutions that allow accurate pipetting into the reaction (∼50–100-fold with respect to the working dilution) and store as single-use aliquots at −80°C.
  a. Right before use, incubate the nuclei suspension for 5 min at 25°C in a thermomixer.
  b. Add one volume (200 μL) of the nuclei suspension (∼5 × 10^6^ nuclei) to the pre-warmed DNase I reaction buffer.
  c. Incubate at 25°C for exactly 8 min. DNase I exposure causes a concentration-dependent shift of intact HMW DNA toward a lower-molecular-weight smear (Figure 1).
    **CRITICAL:** Timing is critical because the dose-response between DNase I concentration and nick frequency is steep in this range. The working concentration should be chosen as the lowest dose that introduces detectable nicking while leaving the bulk of the DNA at HMW, because over-digestion leads to double-stranded fragmentation. An over-digested preparation is shown in the last lane of Figure 1, where nuclei were treated with 12 ng/ μL of DNase I.

**Figure 1:**
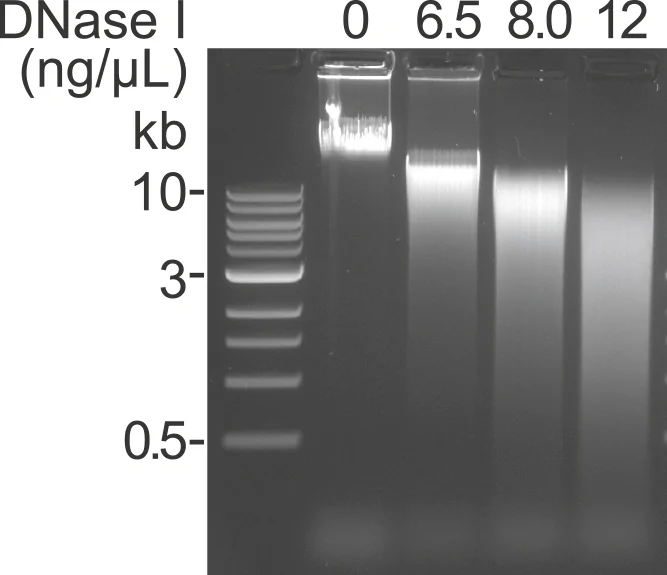
Titration of DNase I treatment in isolated HT1080 nuclei A total of 5 × 10^6^ nuclei from HT1080 cells were incubated with the indicated concentrations of DNase I for 8 min at 25°C. Genomic DNA was then isolated and separated on a 0.8% agarose gel in the presence of ethidium bromide. Increasing DNase I concentration results in progressive fragmentation of genomic DNA, indicating that the treatment conditions can be titrated to introduce DNA damage while retaining high-molecular-weight DNA. Lane M, 1-kb DNA ladder with the 10-kb,3-kb and 0.5-kb bands indicated; lane 1, untreated nuclei (0 ng/μL DNase I); lanes 2–4, increasing DNase I as indicated above each lane. The bracket marks the high-molecular-weight material retained near the well and the arrowhead marks the leading edge of the low-molecular-weight smear. The working concentration selected for this stock (6.5 ng/μL, lane 3) is boxed: it is the lowest dose that produces a detectable smear while retaining the bulk of the signal above 10 kb. Related to steps 16–25.
24. Stop the DNase I reaction immediately by adding 1 volume of ice-cold stop buffer. Mix by inversion three times and place on ice-cold water. The stopped reaction has a final volume of 400 μL and contains approximately 5 × 10^6^ nuclei.

***Note:*** For in vitro genomic DNA treatment as an alternative to nuclei: incubate purified genomic DNA at 0.3 μg/μL in DNase I reaction buffer with 0, 0.1, 0.2 or 0.3 ng/μL DNase I for 8 min at 25°C. Stop with 1 volume of stop buffer and proceed directly to phenol-chloroform extraction (Step 30) without the nuclei isolation steps.

25. Proceed immediately to genomic DNA extraction (step 26). Use the same extraction protocol for DNase I-treated nuclei as for Cas9-induced cell pellets, beginning at Step 26.

### Extraction of high-molecular-weight genomic DNA

**Timing: 2 days. Day 1: Lysis and overnight (12–16 h) proteinase K digestion; Day 2: Extraction, precipitation and resuspension.**

Preservation of HMW DNA is important for successful 2D-gel analysis of i-loops. All manipulations must minimize mechanical shearing. The section starts with either the bulk cell pellet from step 15 or the DNase I-treated nuclei from step 24 and typically yields 20–35 μg of intact genomic DNA per 5 × 10^6^ cells, which is the input for restriction digestion and 2D-gel electrophoresis. Target quality: the bulk of the DNA should migrate as a compact, slowly migrating band well above the largest ladder marker, with no prominent low-molecular-weight smear (Figure 2).

26. Wash the cell pellet from step 15 or the nuclei pellet from step 24, twice with ice-cold PBS. For each wash resuspend gently in 1 mL PBS, centrifuge at 300 × g for 5 min at 4°C and aspirate the supernatant completely.

***Note:*** DNase I-treated nuclei are pelleted at 1000 × g for 5 min at 4°C rather than 300 × g, because free nuclei sediment less readily than intact cells.

27. Resuspend the washed pellet in 500 μL TNE buffer per 5 × 10^6^ cells using a wide-bore pipette tip (prepared by cutting 2–3 mm off the end of a standard 1000-μL tip). Do not vortex at this or any subsequent step.
  a. For cell pellets from step 15: resuspend 5 × 10^6^ cells in 500 μL of TNE buffer. Transfer the suspension in a 15 mL conical tube, for lysis. This is necessary for the following organic extraction step.
  b. For DNase I-treated nuclei from step 24: each final sample contains approximately 5 × 10^6^ nuclei in 400 μl. Add 100 μL of TNE buffer to bring the suspension to 5 × 10^6^ nuclei in 500 μL. Transfer the suspension in a 15 mL conical tube, for lysis.
28. Add an equal volume of prewarmed TNES lysis buffer supplemented with RNase A at a final concentration of 75 μg/mL in the lysis volume. Gently mix by inverting the tube five times. Incubate at 37°C for 45 min.
29. Add proteinase K to a final concentration of 100 μg/mL (from a 20 mg/mL stock). Incubate at 37°C for 12–16 h.

**CRITICAL:** A lysate that is too gelatinous or dense can complicate recovery of the upper phase during organic extraction. If this happens, loosen the lysate by slowly passing it two to four times through a wide-bore tip.

**Pause point:** The proteinase-K-digested lysate can be stored at 4°C for up to 48 h before proceeding to extraction. Do not freeze the lysate at this stage.

30. Perform two sequential phenol-chloroform-isoamyl alcohol 25:24:1 (PCI) extractions.
  a. Add 1 volume (1 mL) of PCI to the lysate.
    i. Cap the tube tightly.
    ii. Mix by gentle inversion 10 times.
      ***Note:*** Do not vortex at any point during or after this step. If the lysate is too dense, the tubes can be incubated for 5 min on a rotating wheel at a low speed (∼10 rpm).
  b. Centrifuge at 12,000 × g for 10 min at 20–25°C. A white denatured-protein interphase will form.
  c. Using a wide-bore pipette tip, carefully transfer the upper aqueous phase to a new 15 mL tube. Avoid touching the white interphase or the lower organic phase. Expect to recover little less than the starting volume, according to the experience of the operator.
  d. Repeat steps a-c once more with 1 ml of fresh PCI if you carried over some protein interphase in the aqueous phase.
    **CRITICAL:** Phenol:chloroform:isoamyl alcohol (25:24:1) is highly corrosive and toxic. Phenol causes severe chemical burns on contact with skin or eyes and can be absorbed transdermally; chloroform is a suspected carcinogen and central nervous system depressant. All manipulations must be performed in a certified chemical fume hood. Wear chemical-resistant gloves (thick nitrile or neoprene; note that standard thin nitrile gloves provide limited protection against phenol), a lab coat and safety goggles. Collect all organic-phase waste and contaminated plasticware in a sealed, labeled halogenated organic solvent waste container for disposal by your institution's safety office.
31. Perform one chloroform extraction.
  a. Add 1 volume of chloroform to the recovered aqueous phase.
  b. Mix by gentle inversion 10 times.
  c. Centrifuge at 12,000 × g for 10 min at 20–25°C.
  d. Transfer the upper aqueous phase to a new tube. Avoid carrying over the lower organic phase.
32. Precipitate the DNA.
  a. Add 1/10 volume of 3 M sodium acetate pH 5.2 to the aqueous phase.
  b. Add 1 volume of isopropanol to the aqueous phase.
  c. Mix by gentle inversion 10 times.

***Note:*** For dense lysates, complete mixing and DNA precipitation may require additional gentle inversions. During this transition, the aqueous phase may become gelatinous. Continue inverting gently until clear white, filamentous gDNA precipitates are visible in an otherwise homogeneous solution.

33. Centrifuge at 16,000 × g for 60 min at 4°C. Aspirate the supernatant carefully without disturbing the pellet.
34. Wash the DNA pellet with 1 volume of 70% ethanol.
  a. Centrifuge at 16,000 × g for 5 min at 4°C.
  b. Aspirate the ethanol completely being careful not to disturb the pellet.
  c. Spin again the tube at 16,000 x g for 10 s and remove any remaining traces of ethanol with a fine-tipped pipette.
35. Air-dry the pellet at 20–25°C for 2–5 min. Add 100–200 μL of 10 mM Tris-HCl pH 8.0 and allow the DNA to resuspend at 20–25°C for 30 min and then at 4°C for 2–18 h without pipetting.

***Note:*** Do not overdry the DNA pellet; a white or cracked pellet indicates over-drying, which makes resuspension difficult.

***Note:*** After initial resuspension, homogenize by gently flicking the tube three to five times or by slowly pipetting up and down with a wide-bore tip. Check extraction quality by NanoDrop spectrophotometer. Expected absorbance ratios A260/A280 ≥ 1.8; A260/A230 ≥ 1.7. Quantify the DNA preparation using a QuBit 4 Fluorometer. Verify HMW integrity by loading 200–500 ng on a 0.8% agarose gel. Intact genomic DNA should migrate as a single tight, HMW band that remains close to the well, with minimal trailing smear toward lower molecular weights (Figure 2B, lane 2). A degraded preparation appears as a broad smear descending well below the main band (Figure 2B, lane 3), while DNA that is poorly resuspended fails to enter the gel properly and produces an irregular band with multiple trails (Figure 2A). Expected yield: 20–35 μg per 5 × 10^6^ cells.

**Pause point:** Purified genomic DNA can be stored at 4°C for up to 4 weeks. Do not freeze the DNA; repeated freeze-thaw cycles can fragment HMW DNA and compromise the resolution of large i-loop-containing molecules in 2D-gels.

**Figure 2:**
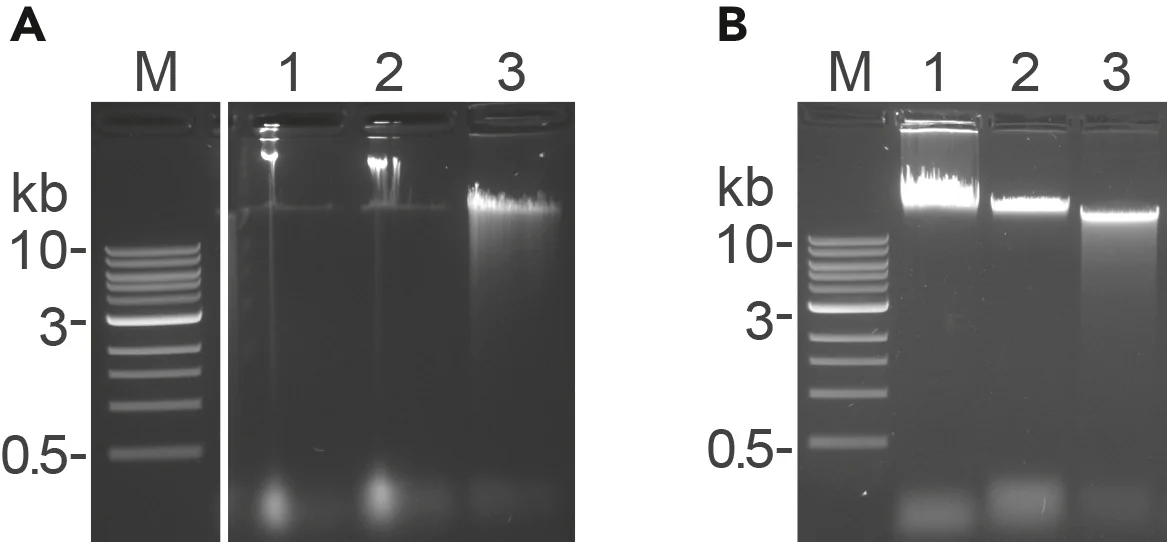
Quality control of genomic DNA preparations (A) Examples of poorly resuspended genomic DNA preparations. Genomic DNA from HT1080 cells was separated on a 0.8% agarose gel and stained with ethidium bromide. Lanes 1 and 2 show HMW DNA retained near the wells, consistent with incomplete resuspension before loading. Lane 3 shows uneven migration and smearing of the HMW DNA, consistent with a partially resuspended or heterogeneous DNA preparation. These preparations should be re-resuspended, not loaded on a 2D-gel. (B) Examples of genomic DNA preparations of different quality separated as in (A). Lane 1 shows an overloaded HMW DNA sample, visible as an intense signal retained near the well. Lane 2 shows an intact HMW genomic DNA preparation, with most of the signal retained above 10 kb and minimal lower-molecular-weight smear. Lane 3 shows a partially degraded genomic DNA preparation, with a lower-molecular-weight smear extending below the main HMW DNA signal. Lane M, 1-kb DNA ladder; the 10-kb, 3-kb and 0.5-kb marker positions are indicated. 200–500 ng of each preparation was loaded. Related to step 35.

### Verification of nick formation by alkaline agarose gel electrophoresis

**Timing: 3 days. Day 1: Plug preparation. Day 2: Gel run. Day 3: Southern blotting and hybridization.**

This procedure is adapted for monitoring nick formation at human telomeric repeats. For a general protocol on alkaline agarose gel electrophoresis, refer to Green and Sambrook, 2021.^16^ Alkaline gel electrophoresis denatures the DNA double helix, separating individual strands and resolving single-strand breaks as a mobility shift toward lower apparent molecular weight. Probing the resulting Southern blot with strand-specific telomeric oligonucleotides allows detection of SSB accumulation on the G-rich or C-rich telomeric strand. This section is the decision point of the protocol: a positive, strand-appropriate signal here confirms that the nickase is active at the target repeat and justifies committing the remaining genomic DNA to the longer 2D-gel workflow. Its input is the agarose-plug aliquot reserved at step 15 (or DNase I-treated nuclei from step 24) and its output is the strand-specific Southern blot shown in Figure 3 (Step 3 of the graphical abstract).

36. Prepare agarose plugs at 1 × 10^6^ cells per plug from cells reserved in step 15 or from the DNase I-treated nuclei suspension (step 24). Prepare a minimum of two plugs per sample, in order to have at least one backup. For 2 × 10^6^ cells:
  a. Pellet cells at 300 × g for 5 min at 4°C, aspirate PBS and resuspend in 120 μL ice-cold PBS.
  b. Prepare 50 mL of 2% low-melting-point agarose in PBS at 50°C. Prewarm the cell suspension for 1–2 min at 37°C and keep the agarose at 50°C.
  c. Quickly mix cells and molten agarose 1:1, 120 μL each, by pipetting three to four times with a wide-bore tip. During mixing, avoid fully expelling the agarose-cell suspension from the tip, as this can introduce air bubbles. Immediately dispense into plug molds, 100-μL wells. Avoid bubble formation by dispensing the agarose mix slowly and evenly.
  d. Allow plugs to solidify on ice for 15 min. Eject solidified plugs into 1.5 mL microcentrifuge tubes. Up to three plugs per sample can be stored in the same tube.
  e. To each tube, add 1 mL of 1× proteinase K buffer, supplemented with 1 mg/mL proteinase K. Incubate tubes on a thermomixer with gentle shaking at 400 rpm, 12–16 h at 50°C.
  f. The next day, carefully aspirate the buffer and rapidly wash with 1 mL of 1× TE buffer. Add fresh 1× TE buffer and incubate with gentle shaking at 400 rpm, at 37°C for 1 h. Repeat the wash three times.
  g. Aspirate the buffer and replace with 1 mM PMSF in 1 mL of 1× TE buffer, to inactivate proteinase K. Incubate with gentle shaking at 400 rpm, at 37°C for 1 h.

**Pause point:** Plugs can be stored at 4°C for up to 1 month if not used immediately for alkaline gel electrophoresis. Do not freeze.

***Note:*** When assessing nick formation, embedding cells in agarose plugs should be preferred to using purified genomic DNA. This minimizes mechanical shear and better preserves the native SSB frequency. Purified HMW genomic DNA can be substituted if plug casting is impractical, but only if it passes the integrity check in Figure 2B; sheared or partially degraded preparations will increase the low-molecular-weight signal, which cannot be distinguished from genuine Cas9- or DNase I-induced breaks.

37. Carefully place one plug per sample in a new 1.5 mL tube. Wash the plugs to remove salts: add 500 μL ddH_2_O per tube and incubate at 20–25°C for 10 min with gentle rocking. Aspirate and repeat once more.
38. Equilibrate plugs in alkaline electrophoresis buffer: replace ddH_2_O with 500 μL alkaline electrophoresis buffer. Incubate for 30 min at 20–25°C with gentle rocking.
39. Prepare 500 mL of 0.8% Low EEO agarose gel in alkaline electrophoresis buffer.
  a. Melt the agarose in 450 mL final volume of ddH_2_O.
  b. Cool the solution by incubating for at least 30 min at 55°C in a water bath.
  c. Add 0.1 volume (50 mL) of 10× alkaline electrophoresis buffer, mix well and immediately pour the gel using wide combs that can accommodate the plugs.
  d. Allow the gel to solidify at 20–25°C for at least 1 h.
  e. Fill the electrophoresis tank with freshly-prepared alkaline electrophoresis buffer.

**CRITICAL:** Do not melt agarose directly in alkaline electrophoresis buffer. Hot alkaline conditions can hydrolyze the agarose polysaccharide, which can distort single-stranded DNA migration during alkaline electrophoresis. Use freshly prepared alkaline electrophoresis buffer for the gel and running buffer and allow the gel to cool fully to 20–25°C before starting the run.

40. Load the plugs in the wells.
  a. Fill the wells with alkaline electrophoresis buffer.
  b. Carefully place each plug horizontally into a gel well using a flat spatula or blunt forceps.
  c. Leave an empty well for loading the DNA marker.
  d. Aspirate the excess alkaline electrophoresis buffer from each well.
  e. Seal the plug in the well with a small volume of molten 0.8% alkaline agarose.
  f. Allow to set for 5 min.
41. Start the run of the gel.
  a. Place the gel in the gel apparatus and connect the peristaltic pump.
  b. Load a 1–10 kb DNA ladder, prepared in alkaline gel-loading buffer (see note below) and initiate electrophoresis at 2 V/cm for 10 min without buffer circulation.
  c. Start the peristaltic pump at a flow rate of 12 mL/min and continue running for 16 h 20 min.

***Note:*** Buffer recirculation is essential to prevent alkaline gradient formation across the gel, which would distort DNA migration.

***Note:*** Prepare the DNA ladder immediately before loading. Dilute an appropriate amount of dye-free 1–10-kb DNA ladder, typically 500 ng per well, in 1× alkaline electrophoresis buffer, then add 0.2 volume of 6× alkaline gel-loading buffer. Mix gently and load into the marker well. Do not use ready-to-load DNA ladders supplied in standard neutral loading dyes, because bromophenol blue is rapidly bleached at high pH and neutral loading buffers do not maintain alkaline sample conditions.

42. At the end of the run, discard the running buffer. Neutralize the gel by submerging it in neutralizing solution for 45 min at 20–25°C with gentle rocking.
43. After neutralization, stain the gel in 0.3 μg/mL ethidium bromide in 10 mM Tris-HCl pH 7.5 for 45 min. Image the gel on a ChemiDoc imager (Figure 3).

***Note:*** An alkaline gel stains markedly more faintly than an equivalent neutral gel. The ethidium bromide signal provides a loading control and an assessment of DNA migration distance and quality. Nick detection is achieved by strand-specific Southern hybridization in Step 44.

**CRITICAL:** Ethidium bromide is an intercalating mutagen and a suspected carcinogen. Always handle ethidium bromide solutions wearing nitrile gloves, a lab coat and eye protection. Prepare and dispose of ethidium-bromide-containing gels and staining solutions in a designated area. Decontaminate liquid ethidium bromide waste before disposal, for example using activated charcoal or a commercial decontamination kit, in accordance with institutional regulations. Do not pour ethidium bromide waste down the drain.

44. Process the gel for Southern blotting as described in “Southern blotting and probe hybridization”, steps 58–68. After UV-crosslinking, hybridize the membrane with strand-specific end-labeled telomeric probes:
  a. The (CCCTAA)_4_ oligonucleotide, labeled with γ-^32^P-ATP, hybridizes to the telomeric G-rich strand (TTAGGG)_n_. Under the conditions described here, this probe is expected to reveal SSBs on the G-strand, induced by the Cas9^H840A^ variant. A low-molecular-weight signal, detected by this probe, in a sample expressing the Cas9^D10A^ variant, might be indicative of DSB formation.
  b. The (TTAGGG)_5_ oligonucleotide, labeled with γ-^32^P-ATP, hybridizes to the C-rich strand (CCCTAA)_n_. Under the conditions described here, this probe is expected to reveal SSBs on the C-strand, induced by the Cas9^D10A^ variant. A low-molecular-weight signal, detected by this probe, in a sample expressing the Cas9^H840A^ variant, might be indicative of DSB formation, which could occur as replication forks run into persistent SSBs.
  c. For alpha satellite-repeat analysis: hybridize with the ^32^P-labeled 316-bp alpha satellite amplicon (probe preparation: “Southern blotting and probe hybridization”, step 63c). This alpha satellite probe is double stranded and can therefore be used to assess Cas9-associated DNA damage at alpha satellite repeats but not strand specificity.

***Note:*** Hybridizing the two strand-specific probes sequentially on the same membrane, with a stripping step in between (steps 69–74), rather than on two separate blots removes eventual variabilities and workload associated with loading and transferring two separate gels.

***Note:*** A positive alkaline gel result supports Cas9 nickase activity at telomeric repeats, before the remaining DNA is committed to 2D-gel analysis. Consistent with the fact that the D10A and H840A nickase variants differ in nick persistence, we typically detect a robust lower-molecular-weight signal with the D10A variant and a much weaker one with the H840A variant (Figure 3). The presence of a low-molecular-weight signal on the non-targeted strand might be indicative of double-strand breaks, which can accumulate when replication forks run into persistent and abundant SSBs.

**Figure 3:**
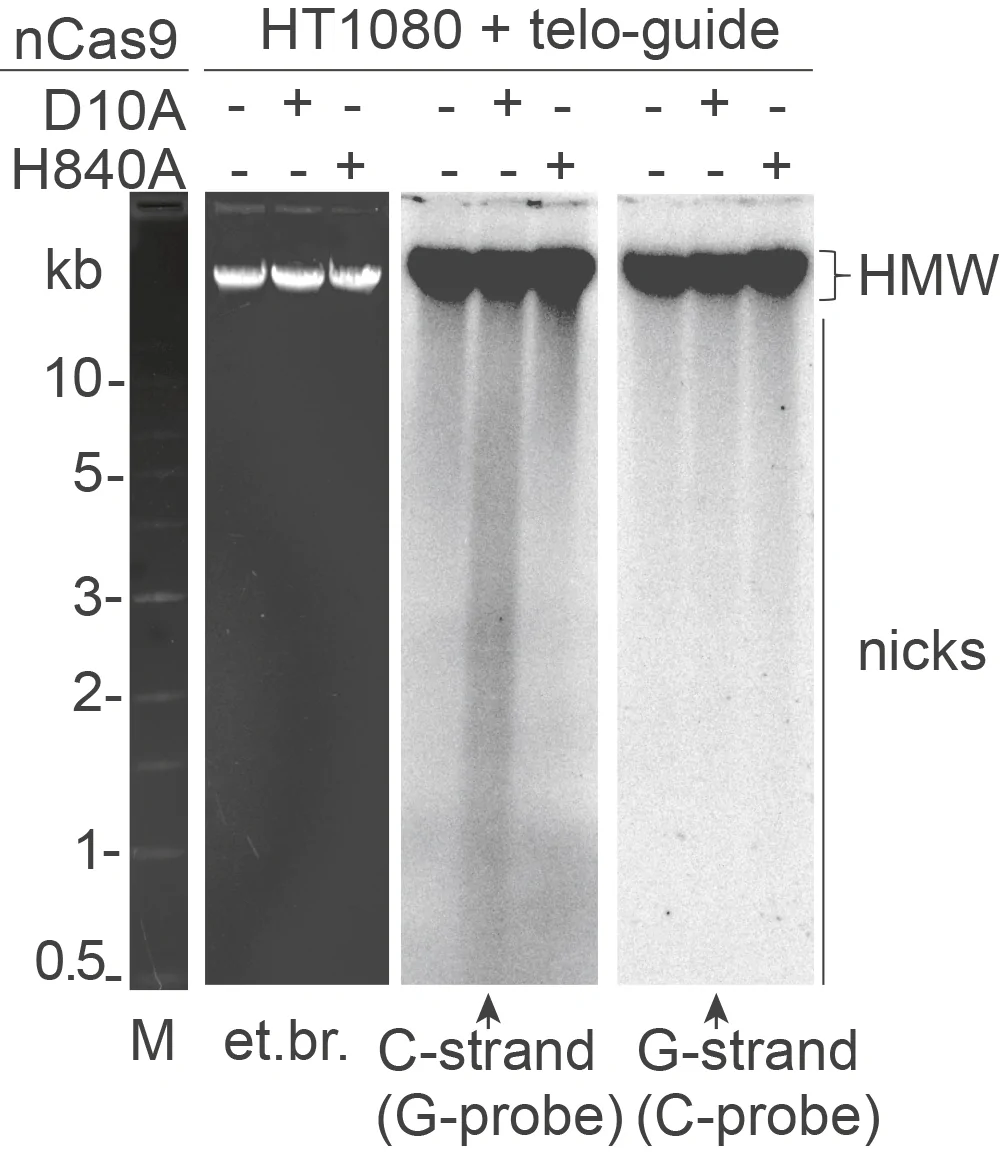
Detection of Cas9-nickase-induced, strand-specific SSBs by alkaline gel electrophoresis HT1080 cells expressing the indicated Cas9 nickase variants were transduced with a telomeric sgRNA (control lane: does not express Cas9 protein). Sixteen hours after doxycycline induction, cells were harvested and genomic DNA was prepared in agarose plugs. Samples were separated by alkaline gel electrophoresis and stained with ethidium bromide, left panel. DNA was then transferred to a membrane and sequentially hybridized with strand-specific 32P-end-labeled telomeric oligonucleotide probes. The (CCCTAA)4 probe recognizes the telomeric G-rich strand, whereas the (TTAGGG)5 probe recognizes the telomeric C-rich strand. Arrows indicate the lanes in which the detected telomeric strand is targeted by the corresponding Cas9 nickase variant. Lane assignments (identical in all three panels): lane M, dye-free 1–10 kb DNA ladder, with the 10-, 5-, 3-, 2-, 1- and 0.5-kb positions marked to the left of the ethidium bromide panel; lane 1, no-guide control pool, doxycycline-induced; lane 2, Cas9D10A + telomeric sgRNA, doxycycline-induced; lane 3, Cas9H840A + telomeric sgRNA, doxycycline-induced. Brackets to the right of each hybridization panel mark the high-molecular-weight telomeric signal and the induced low-molecular-weight signal that reports single-strand breaks on the probed strand. The desirable result is a low-molecular-weight signal in lane 2 with the (TTAGGG)5 probe and in lane 3 with the (CCCTAA)4 probe, with each induced lane compared against control sample. Both hybridization panels were acquired from the same membrane, stripped between probes (steps 69–74), and each was exposed and scanned separately: 12 h for (TTAGGG)5 and 48 h for (CCCTAA)4. Panels are therefore not on a common intensity scale and the two probes must not be compared with each other for absolute signal. The Cas9H840A-induced signal is intrinsically weaker than the Cas9D10A signal because H840A-generated nicks are turned over more rapidly; this difference is reproducible across biological replicates and is a property of the system rather than of the blot. Displayed images are unmodified 16-bit scans with linear display scaling applied identically within each panel; no gamma or local adjustment was used. Source scans are available at Mendeley Data (https://doi.org/10.17632/hwf38mddyt.1). Related to steps 36–44.

### Two-dimensional agarose gel electrophoresis for i-loop detection

**Timing: 3–4 days. Day 1: Restriction enzyme digest. Day 2: First-dimension gel. Day 3: Second-dimension gel. Day 4: Southern blotting.**

2D-gel electrophoresis resolves DNA molecules first by molecular weight and then by shape.^17^ The first-dimension gel is low-percentage, run at low voltage and without ethidium bromide; conditions that minimize the contribution of shape complexity in the migration and therefore separate restriction fragment primarily according to their molecular mass. The second dimension is run at conditions that maximise the contribution of shape complexity in the migration: higher agarose concentration, higher voltage and the presence of ethidium bromide, which increases DNA rigidity. I-loop-containing molecules form a distinct slow-migrating arc above the linear arc on the second-dimension Southern blot; this arc provides a diagnostic readout of i-loop accumulation. The input is 10 μg of restriction-digested genomic DNA from step 35 and the output is a second-dimension gel ready for Southern transfer (Step 4 of the graphical abstract).

45. Digest 10 μg of purified genomic DNA (described in “Extraction of high-molecular-weight genomic DNA”) per condition in a 100-μL reaction volume at 37°C overnight (12–16 h). Always digest the induced and uninduced conditions using restriction enzyme master mixes prepared from the same batch to ensure identical digestion efficiency.
  a. For human telomeric repeats: 20 U HinfI + 20 U RsaI in NEB CutSmart buffer 1×.
  b. For human alpha satellite repeats: 20 U MspI in NEB CutSmart buffer 1×.
  c. Precipitate the digested DNA as described in “Extraction of high-molecular-weight genomic DNA”, steps 32–35 and resuspend in 20–30 μL of TE 1×. Make sure that the wells of the first-dimension gel can easily accommodate all the loading volume.
46. First-dimension gel preparation: cast a 0.35% Low EEO agarose gel (approximately 20 × 25 cm) in TBE 0.5× without ethidium bromide. Allow the gel to solidify for at least 45 min at 4° C. Fill the gel tank with 0.5× TBE without ethidium bromide.

**CRITICAL:** Within the typical induction levels, under the conditions described here, telomeric i-loops are visible by loading at least 10 μg of DNA. If less than 10 μg are recovered after digestion and precipitation, consider digesting a higher starting amount of DNA before proceeding.

47. Load the gel leaving one empty well between samples to facilitate lane excision (Figure 4, top). Load a DNA ladder (HindIII-digested lambda DNA or a 1–10 kb ladder) in a flanking lane that will not be included in the second-dimension slice.

**Figure 4:**
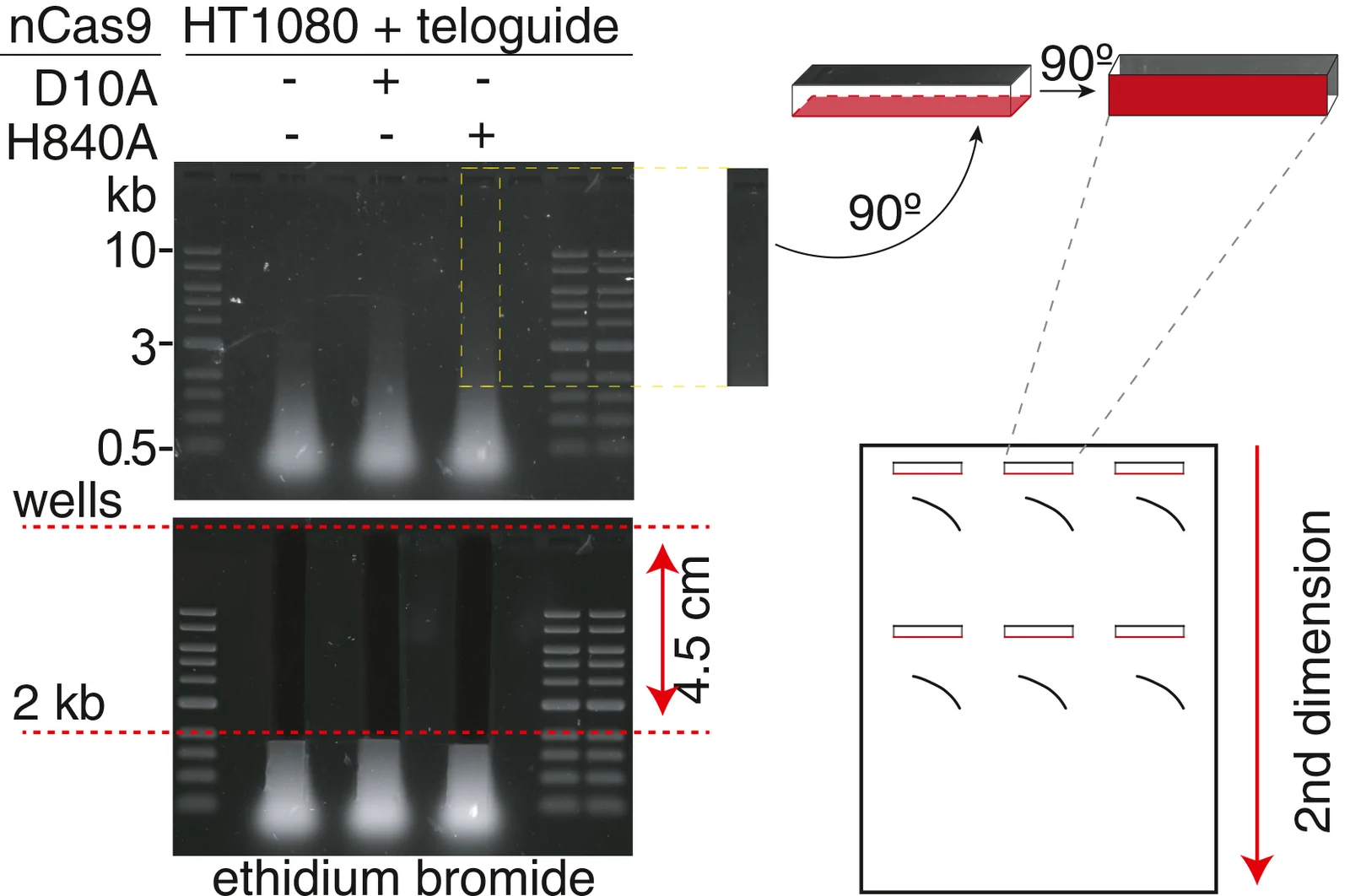
Excision and orientation of first-dimension gel slices for 2D-gel electrophoresis Genomic DNA prepared from cells induced as described in Figure 3 was digested with HinfI and RsaI, separated on a first-dimension agarose gel and stained with ethidium bromide. The top panel shows the first-dimension gel before excision. The bottom panel shows the region selected for second-dimension electrophoresis. For telomeric 2D-gel analysis, each lane is excised from the 2-kb marker position to the region immediately below the wells, thereby retaining high-molecular-weight telomeric restriction fragments while excluding the wells. Horizontal cuts define the size range to be analyzed, and longitudinal cuts separate individual lanes into first-dimension slices. Each slice is then rotated by 90° so that second-dimension migration occurs perpendicular to first-dimension migration. The slice is also oriented so that the smooth surface that was in contact with the tray during the first-dimension gel, shown in red in the schematic, faces the direction of second-dimension migration. Dashed yellow lines indicate the excision cuts; the ladder lane (1-kb, marker positions indicated) is deliberately left outside the excised slice. Related to steps 46–52.
48. Run the first-dimension at 0.5 V/cm for 15 h at 20–25°C (for example 15 V on an apparatus with the electrodes 30 cm apart).

***Note:*** Do not exceed 0.5 V/cm; higher voltage compresses the separation between the linear arc and the i-loop arc in the second dimension.

**CRITICAL:** The first-dimension gel is a low-percentage 0.35% agarose gel and is extremely fragile. Handle with care. Allow sufficient time for the gel to solidify at 4°C before removing the comb. Remove the comb slowly and vertically to avoid tearing the wells. Damaged wells can lead to sample leakage or loss during loading. Immediately before loading the samples, verify well integrity by adding 2–5 μL of 1× loading dye to each well and confirming that the dye remains confined within the well.

49. Stain the first-dimension gel with 0.3 μg/mL ethidium bromide in 0.5× TBE for 45 min at 20–25°C with gentle rocking.
50. Inspect the first dimension gel after it has run.
  a. Visualize the gel briefly on a UV transilluminator. Minimize UV exposure to avoid DNA damage before the second-dimension electrophoresis.
  b. Photograph the gel to document the lane layout and size-marker positions.
  c. Verify that the restriction digest has produced the expected pattern for alpha satellite repeats, a regular repeat ladder with a HMW smear from repeat arrays; for telomeres, a heterogeneous smear.

***Note:*** A substantial accumulation of undigested HMW DNA near the top of the first-dimension gel, visually estimated as >30% of total lane signal above 10 kb, indicates incomplete restriction digestion. This will reduce the resolution of i-loop arcs, because large undigested fragments fail to properly separate in the second dimension. Troubleshooting: see problem 6.

51. Excise the experimental lane from the first-dimension gel using a clean scalpel.
  a. Cut the gel slice as precisely as possible, parallel to the direction of migration (Figure 4, bottom).
    i. For alpha satellite repeats, excise the lane from the 2 kb marker to the well, to retain large repeat-containing fragments.
    ii. For human telomeres excise the region above 2 kb but exclude the well. Handle the slice gently, because 0.35% agarose is extremely fragile.
52. Rotate the excised first-dimension slice by 90° and place it in a new gel tray so that the second-dimension migration will run perpendicular to the first-dimension migration. Place the slice on its side, with the smoothest surface (the one touching the first-dimension gel tray) facing the direction of the second-dimension migration.
53. Second-dimension gel preparation.
  a. Prepare 0.8% low EEO agarose in 0.5× TBE containing 0.3 μg/mL ethidium bromide.
  b. Pour the molten agarose into the casting tray around the rotated first-dimension slices.
  c. Avoid air gaps between the slice and the surrounding agarose and ensure that the slices remain perpendicular to the direction of second-dimension migration.
  d. Allow to solidify for at least 30 min at 20–25°C.
54. Prechill the electrophoresis apparatus and running buffer, 0.5× TBE + 0.3 μg/mL ethidium bromide, to 4°C by placing them in a cold room for at least 1 h before running the second-dimension gel.
55. Run the second dimension at 4.43 V/cm for 3 h at 4°C. Maintain the buffer temperature at 4°C throughout the run by conducting electrophoresis in a cold room.
56. At the end of the second dimension, image the gel using a ChemiDoc or similar imaging system with the ethidium bromide setting to document the position of the major linear arc. This image will serve as a reference for interpreting the subsequent Southern blot.
57. Proceed immediately to Southern blotting (steps 58–68).

### Southern blotting and probe hybridization

**Timing: 2 days. Day 1: Depurination, denaturation and capillary transfer overnight (12–16 h). Day 2: Probe labeling, hybridization, washing and detection.**

Southern blotting transfers DNA from the gel onto a nylon membrane, enabling target-specific hybridization with ^32^P-labeled probes. Radioactive probes are required for the sensitivity needed to detect i-loop arcs, which represent only a small fraction of the total repeat-containing DNA in the gel. This section serves both read-outs of the protocol: it is applied to the alkaline gel (step 44) to visualize strand-specific nicking, and to the second-dimension gel (step 57) to visualize the i-loop arc. Its output is a phosphor-screen image suitable for quantification in step 68.

58. Depurinate the gel to facilitate transfer of large DNA fragments: submerge the gel in depurination solution and incubate for 20 min at 20–25°C with gentle rocking. Discard the HCl solution.
59. Rinse the gel for 2–5 min with ddH_2_O. Denature the DNA by incubating the gel in denaturing solution for 30 min at 20–25°C with gentle rocking. Change the solution and repeat the denaturation step.
60. Neutralize the gel by incubating twice in neutralizing solution for 30 min each at 20–25°C with gentle rocking.
61. Assemble an upward capillary transfer setup to transfer DNA onto a membrane using 10× SSC as the transfer buffer.
  a. Place a flipped gel electrophoresis tray over a buffer reservoir filled with 10× SSC. Lay a sheet of Whatman 3MM paper over the tray with the ends dipping into the buffer reservoir.
  b. Place the gel on the wetted 3MM paper. Flip the gel so that the surface that was in contact with the tray faces upward, because this smoother surface provides better contact with the nylon membrane during transfer. Remove any air bubbles between the paper and the gel by gently rolling a pipette over the gel surface.
  c. Pre-wet a piece of Hybond membrane the size of the gel in 2× SSC for 5 min, then place it on top of the gel.
  d. Cut two additional sheets of Whatman 3MM paper to the same size as the gel. Wet one sheet in 2× SSC and place it on top of the Hybond membrane. Gently roll a pipette over the sheet to remove any air bubble. Add a dry sheet above.
  e. Stack 8–10 cm of dry absorbent paper towels on top of the paper, followed by a flat tray and a 200–500 g weight.
  f. Allow transfer to proceed overnight (12–16 h) at 20–25°C.

***Note:*** After dismantling the transfer setup, mark the membrane with a soft pencil to indicate lane orientation and sample identity. Visualize the gel on a UV-transilluminator after transfer to verify that the DNA has been efficiently transferred (the post-transfer gel should be largely free of ethidium bromide signal in the size range of interest). Troubleshooting: see problem 5.

62. UV-crosslink the DNA to the membrane using a Stratalinker 1800 UV Crosslinker with the auto-crosslink program (1200 μJ/cm^2^ at 254 nm). Immediately proceed to prehybridization with Church mix.
63. Prepare the ^32^P-labeled probe. Three probe types are used depending on the target repeat and probe design:
  a. Double-stranded telomeric probe (Sty11) for standard 2D-gel analysis: prepare 40 μL of template/primer mix by combining 200 ng of the 800-bp EcoRI fragment from pSty11 with 5 ng of the (CCCTAA)_4_ oligo. Denature the template/primer mix at 95°C for 5 min and snap-cool on ice for 2 min. Add 5 μL of OLB buffer, 5 μL of α-^32^P-dCTP (10 mCi/mL) and 2 U Klenow fragment (3′→5′ exo-) and incubate at 20–25°C for 1 h. Purify through a MicroSpin G-50 column following the manufacturer’s instructions. Boil the eluate at 95°C for 5 min and place on ice for 1 min before adding it to the Church mix (Step 65).
  b. Single-stranded telomeric probes for alkaline gel: end-label 50 ng of the (CCCTAA)_4_ or (TTAGGG)_5_ oligonucleotide with 5 μL of γ-^32^P-ATP (10 mCi/mL) and 10 U T4 PNK in 1× T4 PNK buffer at 37°C for 1 h. Add 25 μL of TES buffer to terminate the reaction. Purify through a MicroSpin G-25 column following the manufacturer’s instructions.
  c. Double-stranded alpha satellite probe (316-bp amplicon): amplify a 316-bp fragment from HT1080 genomic DNA using Alpha satellite primer α27 and Alpha satellite primer α30.^10^^,^^11^ Purify the PCR product by gel extraction. Label 50 ng using the Prime-a-Gene Labeling System with α-^32^P-dCTP following the manufacturer’s instructions. Purify through a MicroSpin G-50 column.

**CRITICAL:** Perform all ^32^P work behind appropriate shielding for beta-emitters in a licensed radiation work area. Monitor work surfaces with a Geiger counter before and after each session. Dispose of all contaminated waste, including liquid waste, tips and paper, in designated radioactive waste containers according to institutional regulations.

64. Prehybridize the UV-crosslinked membrane in 25 mL of Church mix in a sealed hybridization tube or cassette using a rotating hybridization oven at 65°C for double-stranded probes or 55°C for single-stranded probes, for at least 30 min.

***Note:*** To ensure uniform hybridization across the entire surface, create a sandwich by placing the membrane between two sheets of hybridization mesh, one sheet on top and one on the bottom. Roll the mesh-membrane-mesh sandwich together before inserting it into the hybridization tube.

65. Proceed with the hybridization.
  a. Discard the prehybridization buffer.
  b. Add the purified probe eluate to fresh Church mix without boiling the Church mix.
  c. Hybridize for at least 4 h or 12–16 h on rotation at the appropriate temperature: 65°C for dsDNA probes or 55°C for ssDNA probes.
66. Remove the hybridization solution and dispose of it as liquid radioactive waste. Wash the membrane three times in the hybridization oven at the hybridization temperature:
  a. Wash 1: 25 mL Church wash (dsDNA probes) - SSC 4× (ssDNA probes), 15 min.
  b. Wash 2: 25 mL Church wash (dsDNA probes) - SSC 4× (ssDNA probes), 15 min.
  c. Wash 3: 25 mL Church wash (dsDNA probes) - SSC 4× + 0.1% SDS (ssDNA probes), 15 min.
67. Discard excess liquid from the membrane, wrap it in plastic wrap and expose it to a phosphor storage screen. Scan using an Amersham Typhoon 5 PhosphorImager.

**Figure 5:**
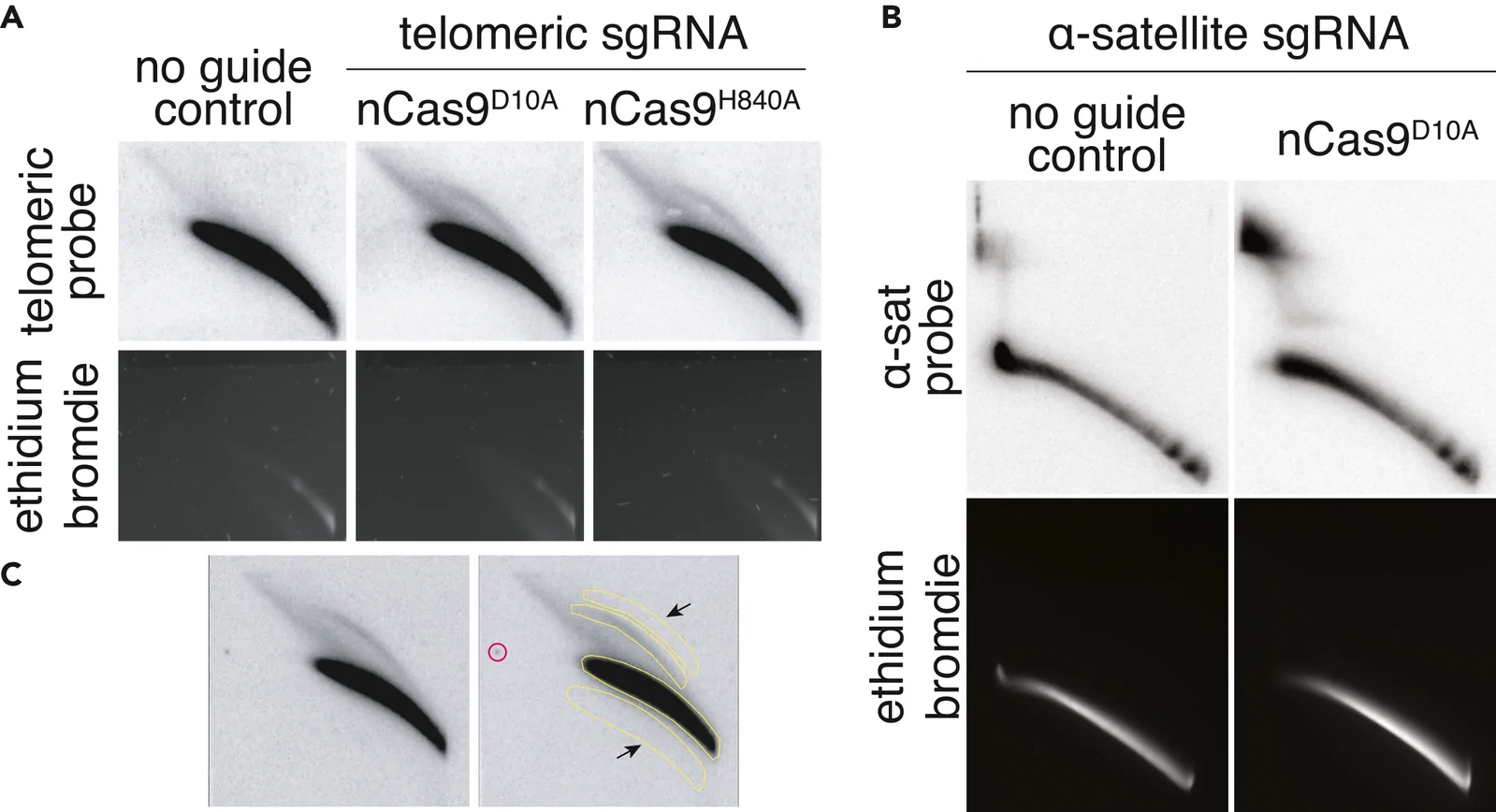
Two-dimensional agarose gel detection of i-loops at telomeric and alpha satellite repeats (A) Detection of telomeric i-loops by 2D-gels. Genomic DNA from cells carrying a telomeric sgRNA and the indicated Cas9 nickase variants was digested with HinfI and RsaI, separated by 2D-gel electrophoresis, transferred by Southern blotting onto a membrane and hybridized with a telomeric probe. The upper row shows repeat-specific Southern hybridization and the lower row shows the corresponding ethidium bromide-stained gels. Linear telomeric restriction fragments migrate as an intense diagonal arc. Upon targeting of Cas9 nickase at telomeres, an additional slow-migrating arc appears above the linear arc, consistent with accumulation of i-loop-containing molecules. This arc is detected in cells expressing telomeric sgRNA together with either nCas9^D10A^ or nCas9^H840A^ and is absent from the no-guide control. Panels from left to right: no-guide control, Cas9^D10A^ + telomeric sgRNA, Cas9^H840A^ + telomeric sgRNA, each doxycycline-induced. The desirable result is a continuous i-loop arc, clearly separated from the linear arc, present in the two nickase panels and absent from the no-guide control. (B) Detection of alpha satellite i-loops by 2D-gels. Genomic DNA from the indicated samples was digested with MspI, separated by 2D-gel electrophoresis, transferred by Southern blotting onto a membrane and hybridized with an alpha satellite probe. The upper row shows alpha satellite-specific Southern hybridization, and the lower row shows the corresponding ethidium bromide-stained gels. In cells expressing the alpha satellite sgRNA together with nCas9^D10A^, an induced slow-migrating signal is detected above the linear arc. This signal is more diffuse than the telomeric i-loop arc, consistent with the larger and more heterogeneous size distribution of alpha satellite-containing restriction fragments. (C) Left, raw scan; right, the same image with quantification ROIs overlaid.Yellow dashed outlines indicate representative regions of interest used to quantify the linear arc and the i-loop arc. Background should be measured from an equivalently sized blank region of the membrane with no DNA signal (arrows). The red circle highlights a nonspecific radioactive spot, which should be avoided when selecting background or signal ROIs.

**Figure 6:**
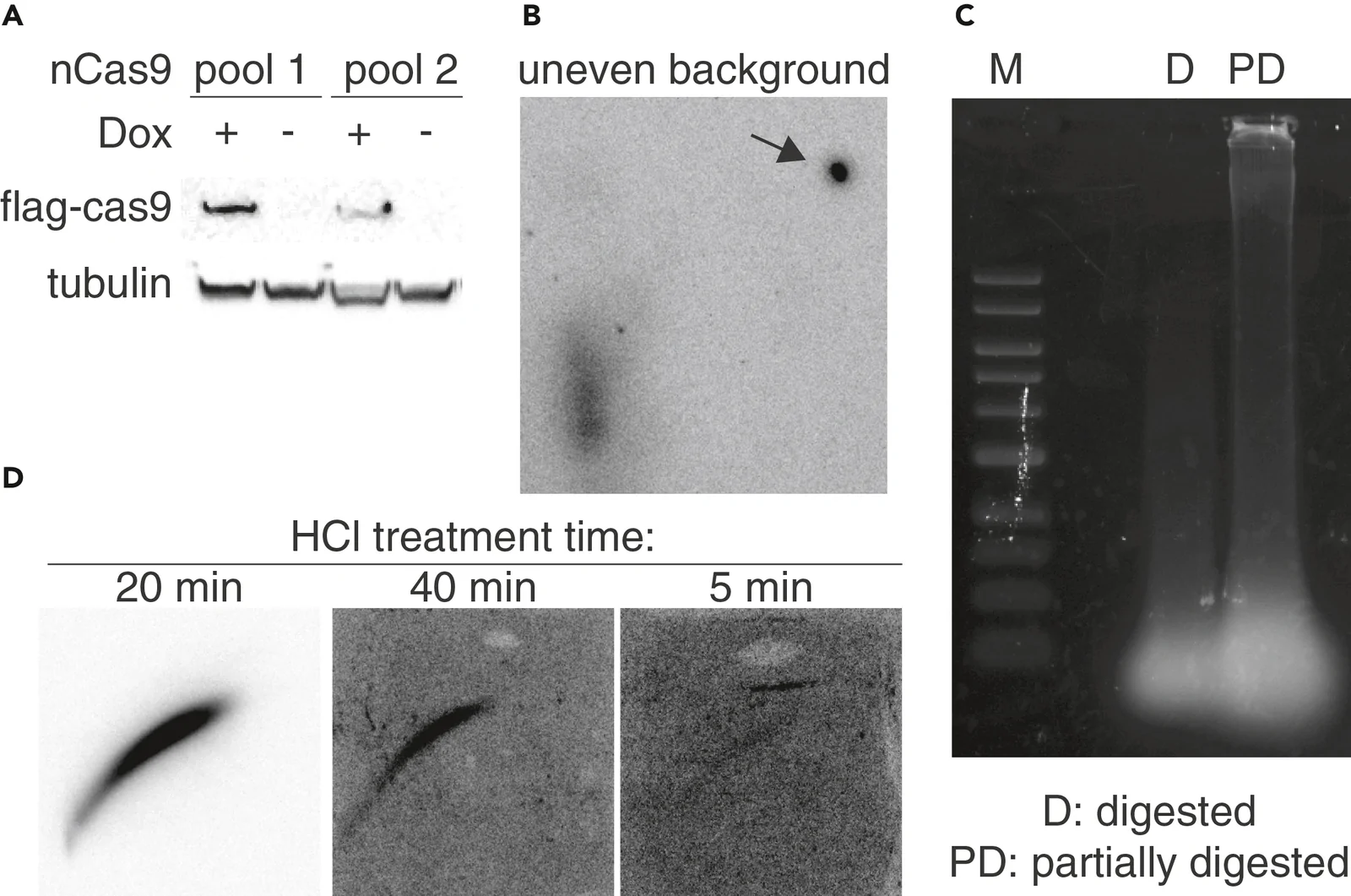
Examples of suboptimal results Outcomes frequently encountered when a step of the protocol goes wrong, provided as a visual reference for the troubleshooting section. Figures 1, 2, and 5 show the corresponding acceptable results. (A) Independently derived cell pools differ in the efficiency of Cas9 induction. Anti-FLAG western blot of two iCas9-Neo pools, each treated with 0.5 μM doxycycline for 16 h (+) or with vehicle alone (−). FLAG-Cas9 (∼160 kDa) is induced in both pools but considerably more weakly in pool 2, while α-tubulin (∼50 kDa) is comparable across all four lanes, excluding unequal loading. Neither pool gives a detectable FLAG-Cas9 signal in the absence of doxycycline. Because the extent of nicking obtained downstream depends on the amount of nickase induced, candidate pools should be compared by western blot at this stage and the pool giving the strongest doxycycline-dependent signal carried forward. Related to step 6 and to troubleshooting problem 1. (B) Uneven hybridization background. Southern blot membrane hybridized with the telomeric probe, showing an unevenly darkened areas across the membrane and a discrete high-intensity spot (arrow) probably caused by probe precipitate. Both features could intefere with arc quantification by introducing non-specific signal inside the regions of interest; a spot of this kind must in any case be excluded from both signal and background selection (step 68). Compare with the acceptable blots in Figure 5A. Related to steps 64–67 and to troubleshooting problem 4. (C) Incomplete restriction digestion. Ethidium bromide staining of a first-dimension gel of genomic DNA digested with HinfI and RsaI. Lane PD, partially digested sample, in which a significant portion of the DNA is retained as high-molecular-weight material near the well; lane D, completely digested sample, in which the DNA is distributed as a heterogeneous smear; lane M, 1-kb DNA ladder. A sample resembling lane PD should be re-digested rather than carried into the second dimension, because undigested high-molecular-weight fragments do not separate well in 2D-gel, preventing the detection of the i-loop arc. Related to step 50 and to troubleshooting problem 6. (D) Example of the consequences of a failed depurination step during Southern blotting. Three samples with the same amount of DNA were separated on 2D-gels and subjected to Southern blotting procedure, with three different conditions of the depurination step. The sample on the left was treated exactly as indicated in the protocol (step 58). The one the middle was incubated for 40 min instead of 20, while the one on the right was incubated for only 5 min. After capillary transfer the membranes were hybridized with a telomeric probe. While the sample in the middle, was probably over-fragmented before the transfer, almost no transferred DNA is visible in the sample on the right.

***Note:*** Typical exposure times are 4–8 h for a strong signal or 24–48 h for weaker signals (e.g., Cas9-induced samples near the detection threshold). On a successful 2D-gel Southern blot, the linear DNA resolves as a smooth diagonal arc and i-loop-containing molecules appear as a discrete second arc migrating above and therefore slower than, the linear arc (Figure 5). The telomeric i-loop arc is well resolved when using either Cas9 nickase variant and is absent from the no-guide control; at alpha satellite repeats the induced signal is present but more diffuse, because the larger and more heterogeneous repeat-containing fragments do not collapse into a single sharp arc (Figure 5B).

***Note:*** If membrane background is high after washing, perform an additional wash for 15 min in SSC 4× + 0.1% SDS at the appropriate hybridization temperature. Figure 6B shows a membrane with an unevenly darkened field and a focal spot of precipitated probe, and Figure 6D shows how declining transfer efficiency raises background and weakens the arcs; Figure 5A is the acceptable reference for comparison. Troubleshooting: see problems 4 and 5.

68. Quantify i-loop and linear arc signals in Fiji/ImageJ.^12^
  a. Open the scanned PhosphorImager image in Fiji. Work on the raw 16-bit scan. If display brightness or contrast are adjusted, do not press apply as that operation modifies pixel values. Confirm that the arcs to be quantified contain no saturated pixels by inspecting the histogram (Image > Adjust > Threshold, or Analyze > Histogram) and checking that the maximum pixel value in the signal region is below the detector maximum. If pixels are saturated, requantify a shorter exposure of the same membrane.
  b. Draw a region of interest (ROI) over the i-loop arc and a second ROI over the linear arc in the same lane (Figure 5C).
  c. Measure the integrated density for each ROI, defined in Fiji/ImageJ as the sum of pixel values within the ROI. Subtract background from an equally sized blank region of the blot with no DNA signal. Because integrated density scales with ROI area, the background ROI must be identical in size and shape to the signal ROI it corrects; duplicate the signal ROI and move it to a blank region rather than drawing a new one.
  d. Calculate the i-loop fraction: [i-loop ROI − background]/([i-loop ROI − background] + [linear arc ROI − background]) × 100. This normalization corrects for differences in total DNA loaded between lanes.
  e. For statistical comparison across conditions, analyze at least three independent biological replicates, defined as independent induction or treatment experiments from cells at different passage numbers. Report mean ± SD. Analyze using a one-tailed Mann-Whitney U test (for two-group comparisons) or one-way ANOVA with Šídák correction (for multiple conditions), as appropriate.

***Note:*** When defining ROIs for both signal quantification (i-loop or linear arcs) and background subtraction, be careful to exclude any non-specific high-signal spots or membrane artifacts. Refer to Figure 5C for an example of proper ROI delineation and an excluded artifactual spot (highlighted in red).

### Stripping and re-probing of radioactive membranes

**Timing: 2–3 h**

In the stripping step, the hybridized probe is washed away by incubation at high temperature in stripping solution, allowing the same membrane to be hybridized with a different probe. This way the two strand-specific telomeric probes can be compared on the same membrane. Use this procedure to hybridize the alkaline-gel blot sequentially with the (CCCTAA)_4_ and then the (TTAGGG)_5_ strand-specific probe (step 44), or to re-probe a 2D-gel blot for a second repeat (step 67). Only strip membranes that were never allowed to dry, as dried probe cannot be removed.

**CRITICAL: (radiation safety):** The membrane and all stripping solutions are radioactive. Perform stripping behind beta shielding in the licensed radiation area, handle the near-boiling solution only with the heat-resistant gloves provided, monitor surfaces with a Geiger counter before and after, and collect the spent stripping solution and washes as liquid radioactive waste per institutional regulations. Do not pour down the drain.

69. After membrane exposure, remove the plastic wrap and rehydrate the membrane in molecular biology-grade water for 5 min at 20–25°C. Keep the membrane wet at all times.
70. Prepare 500 mL of membrane stripping solution (0.1% SDS, 0.1× SSC) and pre-warm to 50–55°C in a water bath.
71. Bring the stripping solution to a boil on a hot plate stirrer, then remove it using the designated heat-resistant gloves and pour it into a container that will fit the membrane.
72. Transfer the membrane into the hot stripping solution and incubate at 65°C with shaking for 30 min to 1 h. Monitor residual radioactivity with a Geiger counter (or a phosphor-screen exposure) until signal is at background.

**CRITICAL:** Do not let the membrane dry between the first hybridization and stripping, or at any point before re-probing; drying irreversibly fixes the probe and prevents stripping and clean re-probing.

73. Once the probe signal is gone, wash the membrane twice with molecular biology-grade water at 20–25°C, 5 min each with gentle rocking.
74. Return the membrane directly to prehybridization in Church mix (Step 64) and proceed with re-probing (Steps 65–67).

***Note:*** Confirm complete stripping by re-exposing the membrane to a phosphor screen before re-probing; residual signal from an incompletely stripped probe will be misread as hybridization by the next probe.

## Expected outcomes

### Western blot: Cas9 nickase expression (step 6)

A clean band at ∼160 kDa should be visible in the doxycycline-induced lane and undetectable or very faint in the uninduced control, consistent with tight Tet-On regulation. GAPDH (∼37 kDa) serves as the loading control. A faint or absent Cas9 band despite doxycycline treatment may indicate that the doxycycline stock has degraded, or that the pool in use induces the transgene inefficiently. In this case one should test a new doxycycline aliquot and, if the signal remains weak, work with a different pool. Independently derived pools can differ appreciably in the efficiency with which Cas9 is induced, as shown in Figure 6A: FLAG-Cas9 is induced strongly in pool 1 and considerably more weakly in pool 2, while the loading control is comparable in both. Because the extent of nicking obtained downstream depends on the amount of nickase induced, we recommend comparing candidate pools by western blot at this stage and carrying forward the one with the strongest doxycycline-dependent signal.

### Alkaline gel electrophoresis: Strand-specific nick verification (steps 36–44)

In cells induced with Cas9^D10A^ and hybridized with the (TTAGGG)_5_ probe a population of low-molecular-weight telomeric signal should accumulate compared to the no-guide control, which retain a HMW telomeric signal. The low-molecular-weight signal should be minimal to undetectable with the (CCCTAA)_4_ probe in the same Cas9^D10A^-induced lane, consistent with strand-specific nick accumulation under these conditions (Figure 3). We consistently see a much weaker accumulation of nicks by the Cas9^H840A^ nickase on the G-strand (Figure 3). This is likely due to a known difference in nick persistence between the two nickases, with the D10A variant generating longer-lived nicks.^18^ However, even the shorter-lived nicks, induced by the Cas9^H840A^ variant, are sufficient to induce i-loops detectable by 2D-gels (Figure 5). Quantitative analysis of the low-molecular-weight signal indicates that, in the experimental setting described here, only a fraction of the telomeres contains one or a few nicks at any given time point.^1^

Although Cas9 nickase activity alone cannot generate double-strand breaks, replication forks encountering persistent SSBs, would lead to double stranded DNA break formation.^19^^,^^20^ Substantial double-strand break formation would be detected as lower-molecular-weight signal on both strand-specific probes or on the probe recognizing the strand not expected to be cleaved by the nickase under the conditions tested. For telomeres, this readout can be used to assess DSB formation, whereas for alpha satellite repeats, where strand-specific hybridization is not possible, a corresponding control requires DNA separation in a neutral gel to assess accumulation of DSBs.

For DNase I-treated nuclei, both strand-specific telomeric probes are expected to show a mobility shift at the treated concentration (6.5 ng/μL) relative to the untreated control (0 ng/μL), as DNase I introduces nicks on both strands without strand specificity. The shift should be concentration-dependent and should be empirically optimized for each DNase I stock (Figure 1).

### 2D-gel electrophoresis for i-loop detection (steps 45–68)

In the uninduced control, telomeric and alpha satellite repeats should generate a linear arc, producing a smoothly curved line with no discernible secondary arc above it. However, a basal level of i-loop signal can be detected in cells with long telomeres or in cells experiencing DNA damage associated with programmed cell death.^1^^,^^21^ Under optimal culture conditions HT1080 cells have a non-detectable background of i-loop structures.

In doxycycline-induced cells, a slow-migrating arc should become visible above the linear arc. This i-loop arc is the diagnostic signature of branched DNA molecules containing internal loops. The induced arc should be specific to the repeat targeted by the active sgRNA, with no corresponding arc induction at the non-targeted repeat in the same cell population. It should be absent in no-guide control and should also be detectable after limited DNase I treatment of isolated nuclei at the same loci, supporting the conclusion that SSBs rather than Cas9-specific effects drive i-loop formation.

Because i-loop containing molecules derive directly from the restriction fragments of the analyzed repeats, the i-loop arc spans this entire range above the linear arc, generally with an increased intensity in the HMW area of the gel. Figure 5A identifies each of these features on the telomeric blot: the position of the wells, the linear arc, the induced i-loop arc, the no-guide control lane and the corresponding ethidium bromide image; Figure 5B does the same for alpha satellite.

Figure 6 collects the suboptimal outcomes we encounter most often and should be consulted alongside Figure 5 whenever a blot is difficult to interpret: uneven hybridization background with focal probe precipitation (Figure 6B), a partially digested first-dimension sample in which a large proportion of the DNA is retained as high-molecular-weight material (Figure 6C), and a series of 2D-gel blots of decreasing transfer efficiency in which the linear arc becomes progressively fainter and the background progressively higher, making the i-loop arc no longer detectable (Figure 6D). In each case the corresponding troubleshooting problem gives the likely cause and the corrective action, together with the criterion for deciding whether the sample can be carried forward.

## Quantification and statistical analysis

Open scanned PhosphorImager images in Fiji/ImageJ. Make sure there are no saturated pixels in the relevant areas to be quantified. Draw regions of interest (ROIs) over (1) the i-loop arc and (2) the linear arc within the same lane. Measure the integrated density for each ROI, defined in Fiji/ImageJ as the sum of pixel values within the ROI, and subtract background measured from an equivalent ROI positioned in a blank region of the membrane. Calculate the i-loop fraction as: [i-loop ROI − background]/([i-loop ROI − background] + [linear arc ROI − background]) × 100%. Perform all quantifications on the raw image without any previous operation that might change pixel values. Figure 5C shows an annotated example of ROI boundaries, correct and incorrect ROI placement and background selection, and step 68 gives a worked calculation using measured integrated-density values.

For statistical comparisons between conditions, for example uninduced versus doxycycline-induced or 0 ng/μL versus 6.5 ng/μL DNase I, use the same tests as in Zanella et al. (2026).^1^ Directional two-group comparisons, in which nick induction or DNase I treatment is expected to increase the i-loop fraction, were analyzed using a one-tailed Mann-Whitney U test. For experiments with more than two conditions, use a one-way ANOVA followed by Šídák’s multiple-comparison correction. Analyze at least three independent biological replicates, defined as independent induction or treatment experiments started from cells at different passage numbers. Report results as mean ± SD. Significance thresholds: *p* < 0.05, *p* < 0.01, *p* < 0.001.

**Note:** The one-tailed Mann-Whitney U test is appropriate only where the direction of the effect is specified in advance on mechanistic grounds, as it is for nick induction. Where no directional prediction applies, for example when comparing two nickase variants with each other, use the two-tailed form.

## Limitations

### Detection size window

2D-gel electrophoresis resolves i-loop structures within DNA fragments of approximately 2–50 kb. I-loops present in fragments exceeding ∼50 kb will not migrate well into the first-dimension and i-loops that are too small to significantly alter the migration of the restriction fragment may co-migrate with the linear arc and remain undetected. This range is well-suited to tandem repeats such as telomeres and alpha satellite, where restriction digestion generates fragments within this window. It is less suitable for detecting i-loops at dispersed repetitive elements, such as LINEs or SINEs, that cannot be released as compact repeat-containing fragments by restriction digestion.

### Restriction-site dependency

The protocol relies on restriction enzymes that generate repeat-containing fragments in the 2–30 kb range without cutting within the repeat unit itself. For telomeres, HinfI and RsaI fulfill this requirement; for alpha satellite, MspI is appropriate under the conditions described here. Detecting i-loops at other repeat classes will require empirical optimization of restriction enzyme combinations.

The absence of suitable flanking restriction sites is a limitation of the restriction-enzyme-based implementation described here rather than of 2D-gel analysis itself. Where no natural enzyme combination releases the repeat in the required size range, custom fragments could in principle be generated by digesting purified genomic DNA in vitro with Cas9 (or another programmable nuclease) ribonucleoproteins directed to unique sequences flanking the repeat, an approach already used to excise defined large genomic segments.^22^ Digestion in plugs would additionally preserve the HMW integrity that the assay depends on. We have not tested this variant, and it would need to be validated carefully: incomplete RNP cleavage would leave undigested HMW material that obscures the arcs, off-target cutting would generate spurious fragments, and residual bound Cas9 would have to be removed by proteinase K treatment before electrophoresis. We note it as a plausible route to extending the method to repeats that lack convenient flanking sites.

### Radioactivity requirement

Southern blotting and probe hybridization rely on ^32^P-labeled probes for the sensitivity required to detect i-loop arcs against the background of bulk genomic DNA. Researchers without access to a licensed radiation facility will need to develop non-radioactive alternatives (e.g., DIG-based chemiluminescent Southern probes), which may require additional optimization to achieve comparable sensitivity.

### Cell-line compatibility

The doxycycline-inducible two-component lentiviral system requires efficient stable lentiviral transduction, antibiotic selection and robust Tet-On regulation. Cell lines with high doxycycline-independent promoter leakiness or cell lines refractory to lentiviral infection, such as certain primary cultures or quiescent cells, may not be suitable. The DNase I alternative is not subject to these constraints and serves as the cell-type-independent option. Transient RNP delivery, discussed in step 9, is a further possible route for such models, although its different induction kinetics would require independent optimization.

## Troubleshooting

### Problem 1

No i-loop arc detected in the 2D-gel Southern blot; only the linear arc is visible (steps 45–68).

### Potential solution

• Cas9 nickase expression may be insufficient, the doxycycline stock may be degraded or sgRNA activity may be poor. Confirm Cas9 expression by anti-FLAG western blot (Figure 6A) and prepare a fresh doxycycline stock. Independently derived pools may differ in induction efficiency, so if the doxycycline-dependent band is weak, compare candidate pools and carry forward the one giving the strongest signal. If necessary, reselect the sgRNA-expressing pool.
• Nick formation may not be detectable by alkaline gel electrophoresis. See problem 2.
• DNA separation in the 2D-gel may be suboptimal, with most of the signal retained in the HMW area of the first-dimension slice. Make sure the DNA sample is fully resuspended and that the restriction digestion is performed under optimal conditions (Figure 6C). Include a DNase I-treated positive control known to contain i-loop structures to confirm that the 2D-gel/Southern workflow can detect the arc.
• An insufficient amount of DNA may have been loaded on the 2D-gel. Re-quantify the DNA and check an aliquot on an agarose gel alongside a control of known concentration.
• Probe labeling or hybridization sensitivity may be insufficient. Repeat hybridization with a freshly labeled probe, increase exposure time and include a positive-control sample.

### Problem 2

No SSBs detected in alkaline gels despite Cas9 expression confirmed by western blot (steps 36–44).

### Potential solution

• Expression of the sgRNA-containing vector may be poor, or guide activity insufficient. Reselect the sgRNA-expressing cell pool under puromycin and confirm the sgRNA sequence.
• Target chromatin may be inaccessible to the Cas9-sgRNA complex. Test an sgRNA targeting an accessible euchromatic unique locus as a positive control for Cas9 activity or test the repeat-targeting sgRNA in another cell line.
• The wrong strand-specific probe may have been used for the nickase variant tested. Confirm the expected D10A/H840A strand specificity and hybridize with the appropriate strand-specific telomeric probe.
• Doxycycline induction time or concentration may be insufficient. Test a limited induction time course or doxycycline concentration range while monitoring for DSB accumulation.
• Alkaline gel denaturation, transfer or hybridization may have failed. Confirm that the gel and running buffer were prepared in alkaline electrophoresis buffer, that the running buffer pH was >12 and that the strand-specific probe gives signal on a positive-control sample. Include a DNase I-treated sample as a positive control.
• The signal may be present but below the displayed exposure. Before concluding that nicking has failed, re-expose the membrane for 24–48 h and scan at an exposure optimized for that probe; this applies particularly to Cas9^H840A^, whose signal is intrinsically weak (Figure 3 and “Expected outcomes”).

### Problem 3

Genomic DNA appears degraded below 10 kb before restriction digest (steps 26–35, quality check; Figure 2B, lane 3).

### Potential solution

• Mechanical shear or harsh handling during extraction, precipitation, washing or resuspension of HMW DNA. Use wide-bore pipette tips, or cut 2–3 mm from standard 1000-μL tips, for all transfers after cell lysis. Never vortex after lysis; mix only by gentle inversion. Avoid overdrying the DNA pellet and resuspend HMW DNA slowly without repeated pipetting.
• DNase contamination of reagents, water, buffers or consumables used during DNA extraction. Prepare fresh buffers using nuclease-free water and clean consumables. Use fresh aliquots of proteinase K, RNase A, TE and restriction-grade water and avoid shared reagent stocks.
• Extensive cell death before or during harvesting, resulting in pre-existing DNA fragmentation. Harvest healthy, subconfluent cultures with minimal floating cells. Avoid over-confluent, nutrient-depleted or apoptotic cultures and assess cell viability before embedding or lysis.

### Problem 4

High or uneven background on the Southern blot membrane after hybridization (steps 64–67, Figure 6B).

### Potential solution

• Post-hybridization washes were insufficient or not stringent enough. Increase wash number, wash volume, wash duration or wash temperature according to the probe and hybridization conditions.
• Probe input was too high or exposure time was too long. Reduce probe input or shorten exposure time; use only exposures within the linear range for quantification.
• BSA or SDS in Church mix was not fully dissolved, causing spotty background and discrete high-intensity spots of the kind indicated with an arrow in Figure 6B. Heat Church mix to 65°C with stirring for ≥10 min before use to dissolve BSA and SDS completely. Use freshly prepared or well-mixed Church mix.
• The membrane was contaminated, allowed to dry, folded or handled improperly during transfer or hybridization. Handle membranes only with clean gloves and clean forceps. Keep the membrane fully wet and submerged during transfer, hybridization and washes, avoid air bubbles or folds and use clean trays, bottles or hybridization tubes.

### Problem 5

Weak or absent Southern signal despite visible DNA in the gel (steps 58–67).

### Potential solution

• Probe labeling efficiency or specific activity is too low. Repeat probe labeling with fresh α-^32^P-dCTP or γ-^32^P-ATP and fresh enzyme. Check labeling efficiency by measuring probe activity before and after column purification.
• The probe was degraded, not denatured before hybridization, or added at insufficient concentration. Denature double-stranded probes immediately before adding them to the hybridization buffer. For oligonucleotide probes, avoid repeated freeze-thaw cycles and prepare a fresh labeled probe if signal is weak.
• The probe was lost during purification, or the wrong purification column was used. Confirm that the purification column is appropriate for the probe size and labeling chemistry. Avoid overloading the column and recover the eluate according to the manufacturer’s instructions.
• Hybridization time was too short, or hybridization temperature and wash stringency were too high for the probe. Extend hybridization to 12–16 h and reduce wash stringency if compatible with probe specificity, for example by lowering wash temperature or increasing SSC concentration.
• DNA was inefficiently transferred to the membrane or was not properly immobilized by UV crosslinking. Verify transfer efficiency by imaging the gel after transfer; the post-transfer gel should be largely depleted of ethidium bromide signal in the size range of interest. Figure 6D shows the effect of progressively poorer transfer on the resulting blot: a weak linear arc against a high background is more often a transfer failure than a hybridization failure. Confirm membrane type, orientation and pre-wetting. Check the bulbs of the UV Stratalinker and the UV-crosslinking settings.

### Problem 6

Incomplete restriction enzyme digestion of genomic DNA; HMW DNA is retained near the well or multiple anomalous arcs are visible in the 2D-gel (step 45, Figure 6C).

### Potential solution

• HMW genomic DNA was not fully resuspended before digestion. Allow HMW DNA to resuspend fully before digestion and mix only by gentle flicking or slow pipetting.
• Residual phenol, SDS, ethanol, salts or EDTA inhibited restriction enzyme activity. If A260/A230 is <1.7, re-precipitate the DNA with sodium acetate/isopropanol and wash twice with 70% ethanol before digestion.
• Restriction enzyme activity was insufficient or reduced. Use sufficient enzyme per μg DNA, add fresh enzyme after the 12–16 h digestion if needed and test enzyme activity on control DNA.

### Problem 7

Double-strand break signal, indicated by accumulation of low-molecular-weight fragments on both strand-specific probes, detected on the alkaline gel (steps 36–44).

### Potential solution

• Doxycycline concentration or induction time is too high, increasing secondary DNA damage or replication-associated conversion of SSBs into DSBs. Reduce doxycycline induction time, for example to 12 h, or reduce doxycycline concentration. If available, compare with a wild-type Cas9 control to define the DSB-associated signal pattern.
• Cells were over-trypsinized, stressed or mechanically mishandled during harvesting. Minimize trypsin exposure, add complete medium immediately after detachment, keep cells cold before embedding and work rapidly from harvest to lysis.

### Problem 8

DNase I treatment of isolated nuclei produces no nick signal on the alkaline gel (steps 16–25).

### Potential solution

• The DNase I stock has low activity because of lot variability, improper reconstitution, repeated freeze-thaw cycles or storage. Reconstitute a fresh aliquot of DNase I and test activity on control DNA, for example by incubating 1 μg lambda DNA with DNase I and confirming degradation on a 1% agarose gel.
• DNase I concentration is too low for the nuclei preparation. Titrate the DNase I concentration for each new enzyme stock or nuclei preparation (Figure 1).
• CaCl_2_ or Mg^2+^ was omitted from the DNase I reaction buffer. Confirm that both were added.
• Incubation time or temperature was incorrect. Perform the reaction for the specified time and temperature and stop the reaction promptly.
• Alkaline gel denaturation failed. Confirm the alkaline electrophoresis buffer composition, verify pH >12 with indicator paper and ensure that the gel and tank were not accidentally prepared or filled with TBE.

### Problem 9

DNase I-treated nuclei show extensive DNA degradation or loss of HMW DNA (steps 16–25).

### Potential solution

• DNase I concentration was too high for the enzyme stock or nuclei preparation. Reduce the DNase I concentration and titrate a lower range.
• Incubation time was too long or the reaction was not stopped promptly. Keep the incubation time fixed and add stop buffer promptly.
• Nuclei were damaged during isolation, increasing uncontrolled nuclease accessibility. Confirm nuclei integrity by phase-contrast microscopy after Triton X-100 treatment.

### Problem 10

An i-loop arc is detected in the uninduced control at levels similar to the induced sample (unexpected baseline signal) (steps 45–68).

### Potential solution

• Elevated cell death or apoptosis in the population due to mishandling, over-confluency, nutrient depletion or improper culturing conditions. Harvest only healthy, actively proliferating cultures. Avoid over-confluent, nutrient-depleted, stressed or apoptotic cultures and minimize trypsin exposure and mechanical handling during harvest. If apoptosis-associated background is suspected, test whether a pan-caspase inhibitor reduces the uninduced i-loop signal.
• Excessive DNA nicking or damage introduced during cell harvesting or DNA preparation. Improve sample handling during harvesting and DNA preparation: work rapidly, keep samples cold when appropriate, avoid harsh pipetting and use wide-bore tips after lysis.
• The cell line has a high intrinsic background of i-loop formation, for example because of very long telomeres or more abundant target repeats. Reconsider the cell line of choice based on your experimental needs.
• Ongoing endogenous DNA damage or replication stress at the time of harvest. Reduce endogenous stress before harvest by optimizing seeding density, medium freshness, treatment timing and cell passage number.
• Leaky doxycycline-independent Cas9 nickase expression, or residual tetracycline-like activity in the FBS lot. Test uninduced cells by anti-FLAG western blot to confirm absence of Cas9 nickase expression. If Cas9 is detected without doxycycline, use a less leaky cell pool or clone and switch to a certified tetracycline-free FBS lot if needed.

## Resource availability

### Lead contact

Further information and requests for resources and reagents should be directed to and will be fulfilled by the lead contact, Elia Zanella (elia.zanella@ifom.eu).

### Technical contact

Technical questions regarding execution of this protocol should be directed to the technical contact, Ylli Doksani (ylli.doksani@ifom.eu).

### Materials availability

Plasmids generated in this study (Lenti-iCas9^D10A^-Neo, Lenti-iCas9^H840A^-Neo, Teloguide, Alphaguide, and α-satellite pSP73) are available upon reasonable request from the corresponding author (ylli.doksani@ifom.eu).

### Data and code availability

This protocol does not generate dedicated bioinformatics datasets. Original source data and images for the associated research paper are deposited at Mendeley Data (https://doi.org/10.17632/hwf38mddyt.1).

## Acknowledgments

The authors thank the IFOM Cell Biology Unit for assistance with cell-line handling and authentication. The Doksani laboratory is supported by the Associazione Italiana per la Ricerca sul Cancro (AIRC IG 28954), the Concern Foundation, and Worldwide Cancer Research (WWCR 24-0166).

## Author contributions

E.Z. developed and optimized the experimental protocol and performed all experiments. E.Z. and F.B. wrote the manuscript draft. Y.D. conceived the experimental strategy, supervised all work, and finalized the manuscript.

## Declaration of interests

The authors declare no competing interests.
